# Combining Experimental and Theoretical Tools to Probe Radio-Oxidation Products in Polyethylene

**DOI:** 10.3390/polym15061537

**Published:** 2023-03-20

**Authors:** Muriel Ferry, Yunho Ahn, Florian Le Dantec, Yvette Ngono, Guido Roma

**Affiliations:** 1 Université Paris-Saclay, CEA, Service de Physico-Chimie (SPC), 91191 Gif sur Yvette, France; muriel.ferry@cea.fr (M.F.); ledantec.florian@gmail.com (F.L.D.); 2 Université Paris-Saclay, CEA, Service de Recherches en Corrosion et Comportement des Matériaux, SRMP, 91191 Gif sur Yvette, France; dbgh1417@naver.com; 3 Normandie Univ, ENSICAEN, UNICAEN, CEA, CNRS, CIMAP, UMR 6252, BP 5133, Cedex 05, F-14070 Caen, France; yvette.ngono-ravache@cea.fr

**Keywords:** polyethylene, radio-oxidation, infrared spectroscopy, carbonyl, density functional theory, ab initio

## Abstract

Polyethylene is one of the most used polymers in a variety of sectors. A typical technique used to assess aging is infrared spectroscopy. Under oxidation, the region of the spectrum that is most studied is the one containing the carbonyl signature. However, various carbonyl groups contribute to the carbonyl peak: ketones, aldehydes, esters, lactones, carboxylic acids, and more. A usual procedure to quantify each of them is the deconvolution of experimental peaks based on experimental assignments of infrared bands. In this paper, we complement this procedure, applied to two polyethylene types, with extended density functional theory (DFT) calculations of infrared spectra, using a polyethylene model mimicking the main features of a semicrystalline polymer. We compare theoretical frequencies and infrared intensities with parameters extracted from the literature that are used to, eventually, estimate concentrations. We provide an alternative estimation entirely based on theoretical data, showing that DFT can be a valuable tool to analyze, or at least complement, experimental data to assess polymer aging. The comparison of different deconvolution procedures raises the question of the contribution of conjugated ketones in the global carbonyl buildup, as well as that of ketones/alcohols pairs, or the relative concentration of esters and aldehydes.

## 1. Introduction

One of the most produced polymers is polyethylene in its various forms (HDPE, LDPE, LLDPE), constituting roughly one-fourth of total annual world plastic production [1]. The domains of applications span from everyday life food packaging, to electric cable insulation, to more niche, though important, sectors like the medical one (e.g., hip protheses). Polyethylene is vulnerable to oxidation, and concerns regarding its use depend on the application, its expected lifetime, and the environmental conditions in which it is used. For example, the oxidation of PE in contact with biological tissues is a concern for medical applications [2]. Harsh environments (temperature, irradiation) constitute a threat for the lifetime of electric cables used in, e.g., nuclear power plants [3] (NPP).

We focus here on model materials similar to those used for the insulation of electric cables in NPP in order to investigate the accumulation of oxidation defects under γ irradiation at doses typical of NPP aging. Even model materials, in general, also contain additives like phenolic antioxidants, whose role is to delay the oxidation of the material. Understanding the role of the pristine microstructure on the aging mechanism is important. For this reason, we choose to compare LLDPE (linear low density polyethylene) with a crosslinked polyethylene (XLPE). The investigation conditions are, for relatively high doses, representative of the environment of NPP [3,4] and, for dose rates, correspond to accelerated aging experiments currently available [5,6,7,8].

Several experimental techniques have been used in order to characterise the accumulation of oxidation species, in particular carbonyl species, in oxidized polyethylene. Electronic paramagnetic resonance (EPR), for example, has been used since the early days of irradiation studies of hydrocarbons to detect transient radical species [9] and has been a very useful tool to characterise some radicals [10,11,12,13]. It is, however, a technique insensitive to most stable final products, which do not carry an unpaired spin. Other optical techniques can be used to probe carbonyl species, for example, photoluminescence (PL) [14,15] or Raman spectroscopy, but they are hardly able to distinguish the various carbonyl species that might be present in oxidized polyethylene from one another.

Other techniques like NMR [16,17] have been used; however, their sensitivity is much lower than what can be considered the technique of choice for a fine characterization of carbonyl species: Fourier transform infrared spectroscopy (FTIR) [18,19,20,21,22,23]. This technique allows for the detection of newly formed species in particular in the regions of 3000–3600 cm${}^{-1}$ (hydroxyl, hydroperoxides) and 1600–1800 cm${}^{-1}$ (carbonyl and insaturations, C=O/C=C bonds). In spite of a lot of remarkable work, the deconvolution of all contributions to, e.g., the experimental signature of carbonyls is far from being straightforward.

Even this powerful tool suffers from limitations related, on the one hand, to the choice of the carbonyl species to be inserted in the deconvolution procedures and, on the other hand, to reliable assignment of characteristic frequencies and absorption coefficients of each of the included chemical groups [23]. One of the difficulties is obtaining reference data that are representative of the local atomic environment; the latter is not even clearly defined, given the fact that samples of polymers to analyze contain amorphous and crystalline phases. Moreover, even excluding the possibility of finding carbonyl species in the crystalline regions—on the basis of the usual assumption that oxygen cannot diffuse through the crystal—frequency and absorptivity can change according to their localization in the amorphous region and/or at the interface between the crystalline and the amorphous regions.

Our goal in this work is to complement experimental characterization by FTIR with theoretical calculations of infrared spectra based on density functional theory (DFT). Calculation of harmonic vibrational frequencies in solids and molecules is based on the calculation of the second derivative of the total energy vs. displacements either by finite differences or by linear response approaches [24]. For molecules, extensive tests with various levels of theory suggest that applying appropriate scaling factors it is possible to correct errors in frequencies that, nevertheless, only rarely exceed a few percent [25]. Calculations of phonon frequencies in inorganic solids are in general within the same error range with respect to experiments.

Intensities associated with a given eigenmode depend on the derivative of the dipole moment with respect to the normal mode coordinate [26]. They can be calculated using DFT for both molecules and solids through Born effective charges and the phonons’ eigenvectors. Benchmarks for a set of small molecules show that DFT gives results comparable to higher level quantum chemistry approaches at a much lower cost [27]. As far as we know, no attempt was made to use IR frequencies and intensities calculated with DFT in order to unravel experimental spectra of carbonyl species in aliphatic polymers; however, a dynamic approach has been applied to uracil [28] and to hydroxyl-containing minerals in order to rationalise their behaviour under pressure [29].

In this paper, we combine DFT calculations of frequencies and IR intensities of various carbonyl species in a model mimicking the interface between crystalline and amorphous phases in PE, with experimental characterization of γ-irradiated PE (LLDPE and XLPE) using FTIR. In Section 2, we give experimental (Section 2.1, Section 2.1.1, and Section 2.1.2) and theoretical (Section 2.2) details of our approach. Then, we present the results in Section 3 where, after giving the main direct outcome of the experiments (Section 3.1) and of the calculations (Section 3.2), we compare and combine them to obtain the evolution of the concentration of various carbonyl species in the two sets of materials (Section 3.3). In the discussion accompanying the presentation of the results (Section 3), we highlight the advantage of combining theory and experiment in this kind of characterization and underline some open questions that arise from our comparison.

## 2. Materials and Methods

### 2.1. Experimental Setup

#### 2.1.1. Sample Preparation

Linear low-density polyethylene (LLDPE), with a density ρ = 0.918 g·cm${}^{-3}$, was either only shaped as films or crosslinked using 1 phr of dicumyl peroxide (DCP). The first step consisted of producing a homogeneous mixture of the polymer and of the crosslinking agent. Mixtures were made in a Haake thermoScientific twin-screw extruder, with the cells having a total volume of 69 cm${}^{3}$. They were prepared under air by heating the cells up to 110 °C and using a 50 rpm blade rotation speed, filling the Haake with LLDPE. After the melting of LLDPE, the crosslinking agent was introduced, and the mixture is left 5 min before removal of the twin-screw extruder. Crosslinking and shaping as films were performed in one single stage in a hydraulic press Polystat 200 T heater from Servitec. The following protocol was applied for both pure LLDPE and for LLDPE + DCP: the material was heated up to 170 °C, then introduced into a 250 μm spacer, which was itself introduced into the hydraulic press until temperature homogenization was achieved. A pressure of 50 bars was applied for 30 s, then it was increased up to 200 bars by 50-bar steps. After keeping the polymer for 15 min at 200 bars, the film was finally removed from the hydraulic press and allowed to cool down before collecting the sample. Hereafter, pure polymer is referred to as LLDPE, whereas LLDPE + DCP is referred to as XLPE. Characterization of both materials was carried out, and the outcomes are described in the Supplementary Information. The following summary can be given: the LLDPE base material contains a small proportion of Irganox 1076, and its gel fraction is null even after shaping. XLPE contains the same primary antioxidant but also DCP decomposition products; its gel fraction is equal to 67 ± 1% before irradiation.

#### 2.1.2. Irradiation

To perform irradiations in controlled atmospheric conditions, each sample was introduced into a glass container of known volume. Ampoules were evacuated using a vacuum line and then filled with reconstituted air (20.0% O${}_{2}$, 77.99% N${}_{2}$, 2.01% Kr), with krypton being used as a tracer to determine the final pressure. Pictures of similar samples in their glass containers can be found in a previous paper [30]. At the end of the irradiation, sample masses were estimated to contain a final H${}_{2}$ content of about 1 vol%. This protocol was performed to allow for quantification of the radiolysis gases: the results, outside of the scope of this article, will be presented elsewhere. γ irradiations were performed using ${}^{60}$Co sources at the Poseidon facility of LABRA (Saclay, France). Dosimetry was performed using a UNIDOS PTW dosimeter equipped with a calibration chamber adjusted every two years by the CEA’s LNHB laboratory. No electronic correction was made to take into account the electronic density difference between water and the polymers. Uncertainties for given doses are less than 6%. Dose rates and doses at which FTIR spectra were collected are gathered in Table 1.

As irradiations were performed under an oxidative atmosphere, the critical thickness not to be exceeded to ensure homogeneous oxidation conditions through the polymer thickness, e${}_{c}$, has to be estimated. We used the Gillen and Clough [31] relationship, given by

(1) $$e_{c}=\sqrt{\frac{8\cdot p\cdot P(O_{2})}{G(-O_{2})\cdot I}},$$

where $P(O_{2})$ is the oxygen pressure (in atm) in the irradiation cell, *p* is the oxygen permeation in the polymer (in mol·cm${}^{2}\cdot$kg${}^{-1}\cdot$atm${}^{-1}\cdot$s${}^{-1}$), *I* is the dose rate (in Gy·s${}^{-1}$), and $G(-O_{2}$) is the oxygen consumption radiation chemical yield (in mol·J${}^{-1}$). $e_{c}$ was estimated to be about 150 μm. Since nominal film thicknesses are about 250 μm, it therefore has to be assumed that irradiations are not performed homogeneously as far as the oxidation is concerned.

#### 2.1.3. Fourier Transform Infrared Spectroscopy

Fourier Transform Infrared (FTIR) spectra of the polymers were acquired using a Bruker Tensor 27 spectrometer equipped with a Bruker Platinum single reflection diamond attenuated total reflectance (ATR) accessory and a DTGS (deuterated triglycine sulfate) detector. Spectra were recorded between 4000 and 600 cm${}^{-1}$ at a resolution of 2 cm${}^{-1}$, with 64 accumulated scans.

Deconvolution is one of the two techniques usually employed to discriminate the absorption bands linked to different functional groups that form a single broad infrared peak [32]. In this study, the fitting was performed on the carbonyl broad peak that grows in PE during radio-oxidation. The fitting was performed assuming the FTIR band assignments given in Table 2, allowing a certain degree of freedom for the positions of the peak maxima and widths.

Deconvolutions were carried out for both materials, but only at the two higher doses listed in Table 1, as the carbonyl signal at 12 and 24 kGy was too weak to obtain reliable results. The baseline to be removed was determined in the area 1650–1800 cm${}^{-1}$. The fitting function used is a combination of Gaussian functions; this choice corresponds to the assumption that linewidths stem essentially from disorder, i.e., that new bonds are formed mainly in the amorphous phase.

For each set of data, we carried out four alternative deconvolutions with different constraints on the FWHM of the Gaussian components. The reason for that is, first, to check how the quality of the fit depends on this parameter and, second, to relate this parameter to the spread of the frequencies obtained theoretically while varying the local environment of each of the considered chemical species. The constraints on the FWHM, assumed to be the same for all chemical groups, were: 5–18 cm${}^{-1}$, 10–20 cm${}^{-1}$, 15–25 cm${}^{-1}$, and 20–30 cm${}^{-1}$. Experimental analysis generally assumes infrared band widths do not exceed 15 cm${}^{-1}$. However, our theoretical results suggest that bands can be larger. Further details are provided in the Supplementary Information.

In addition to the deconvolutions carried out with experimental reference frequencies, a deconvolution of the experimental results was performed with the frequencies obtained by DFT calculations discussed later. In this case, the peaks’ central position were given the same degree of freedom as for experimental reference frequencies, while the widths of the Gaussians were allowed to span the range 10–20 cm${}^{-1}$.

In the following, we will show only the result of one deconvolution (the one with constrained FWHM between 10 and 20 cm${}^{-1}$), but further details are given in the Supplementary Information.

### 2.2. Theoretical Approach

**Figure 1:**
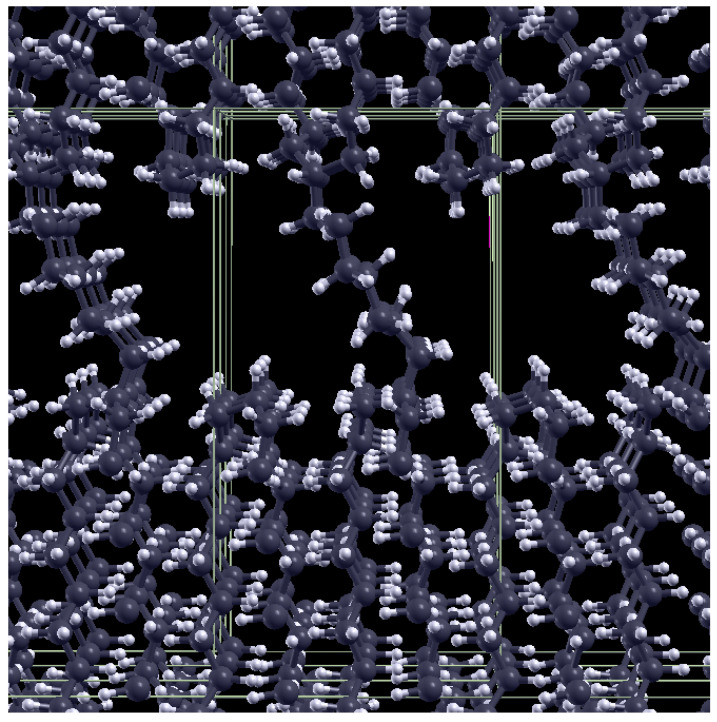
The PE model used for DFT calculations; the periodically repeated unit cell, including a thin crystalline lamella with two surfaces connected by an alkyl chain, is shown. The parameters of the orthorhombic cell, containing 151 atoms, are 9.72, 6.96, and 19.96 Å.

Our theoretical approach to the infrared spectrum of polyethylene containing carbonyl defects is based on density functional theory (DFT). Before describing the technical details of the theoretical framework, we discuss the model used for polyethylene in order to represent both the crystalline and the amorphous regions, as well as their interface. We relied on a slab supercell including a thin crystalline lamella separated by its periodic images, in the direction perpendicular to the lamella surface, by a region that is empty space except for a single chain connecting the two lamella surfaces. This model, containing 150 atoms, is a variant of the model described in detail in [37]; the modification consists of the connecting chain, which was branched on each surface by removing a hydrogen atom. A view of this model can be seen in Figure 1. The model was fully relaxed by keeping fixed the in-plane lattice parameter of the orthorhombic crystal and, for the out-of-plane lattice parameter, we kept the value used in [37]. We note that this gives an overall density (0.88 g/cm${}^{-1}$) that is close to typical experimental values for LLDPE.

Total energy calculations were performed with norm-conserving pseudopotentials and a plane wave basis set with a kinetic-energy cutoff of 80 Rydberg. A Γ-centered 2 × 3 × 2 Monkhorst–Pack **k**-point grid was used for representing screening. The atomic structures were relaxed down to a force threshold of 10${}^{-4}$ Rydberg/Bohr. The chosen exchange correlation (xc) functional was the optB86b van der Waals functional [38], providing a satisfactory description of dispersion forces in polyethylene [39].

The model’s eigenfrequencies and infrared activities were calculated in the framework of density functional perturbation theory (DFPT) [24] using the ph.x module of the Quantum-Espresso software package [40]. The infrared activity for mode ν, $I_{\nu }$, was obtained from the DFPT Born effective charges Z${}_{\alpha ,\beta }^{i}$ and phonon displacements $u_{\nu ,\beta }^{i}$ as

(2) $$I_{\nu }=\sum_{\alpha }{\left|\sum_{i,\beta }Z_{\alpha \beta }^{i}u_{\nu ,\beta }^{i}\right|}^{2},$$

where α and β are cartesian directions and *i* is the label of the atom in the cell. We note that this quantity, proportional to the contribution of a single mode to the IR absorption cross sections ($\sigma_{IR}\left(\nu \right)$), is closely related to the molar extinction coefficient, indicated by $\epsilon_{IR}\left(\nu \right)$, through $\sigma_{IR}\left(\nu \right)=log\left(10\right)\times 10^{3}/N_{A}\times \epsilon_{IR}\left(\nu \right)$, where $N_{A}$ is the Avogadro number.

**Figure 2:**
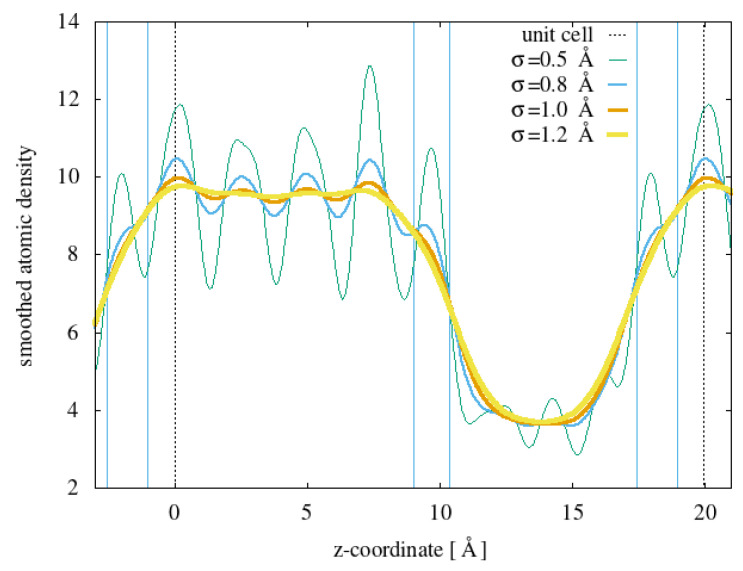
Planar average atomic density along the *z* direction (perpendicular to lamella surfaces) for the model shown in Figure 1. The various curves have been obtained by making different choices for the width σ for the Gaussian function representing each atom. The region considered ”surface” in the following is delimited by blue vertical lines. Dotted lines are the boundaries of the periodic cell.

Carbonyl defects of various types were introduced one at a time into the lamellar model just described at various possible positions; for example, for carboxylic acids, the insertion procedure (somewhat more complex than for other groups) consisted of taking three consecutive carbons, saturating the first with an additional hydrogen, removing the second with the two attached hydrogens, and, for the third, substituting the two attached hydrogens with an oxygen atom and an OH group. After the insertion of the carbonyl group, the structure was then fully relaxed before performing phonon calculations. In order to classify the different insertion sites, the crystal bulk, the surface, and the amorphous region separating the two surfaces were determined on the basis of the in-plane averaged atomic density, as shown in Figure 2.

### 2.3. Calculation of Carbonyl Concentrations

The concentration *c* of the various carbonyl species in the four PE samples has been extracted from the Gaussian height, or the outcome of the deconvolution, through the Beer–Lambert law (A=ϵlc), where ϵ is the molar extinction coefficient at the relevant frequency, assuming that the heights are proportional to the absorbance *A*. The path length *l* corresponds to the effective penetration depth.

FTIR-ATR spectra were recorded as attenuated total reflectance. Using this kind of module, the depth of penetration d${}_{p}$ and the effective penetration d${}_{e}$ can be calculated according to the equations in ref. [23], using the refractive index of the ATR crystal (diamond, n = 2.4) and the refractive index of polyethylene (assuming the value of the pristine material, n = 1.5), plus an incidence angle of 45°. In our case, the calculated effective penetrations are on the order of 2.5 μ for the frequencies of interest, but we calculated them explicitly as a function of the frequency, thus taking into account the slight difference in penetration depth of the beam at different frequencies. We note that the effective penetration depth is then markedly smaller than the critical thickness for oxygen diffusion mentioned above, thus assuring the homogeneity of the oxidation in the analyzed region.

For the ϵ values, those that were not available from experiments were supplied by theoretical values extracted from our calculated IR activities (see Section 2.2), assuming, for a given species *i*, $\epsilon_{i}=\epsilon_{ketone}^{exp}\times \frac{\epsilon_{i}^{theo}}{\epsilon_{ketone}^{theo}}$. The used values will be presented in Section 3.2.

## 3. Results and Discussion

### 3.1. Analysis of Experimental FTIR Spectra

**Figure 3:**
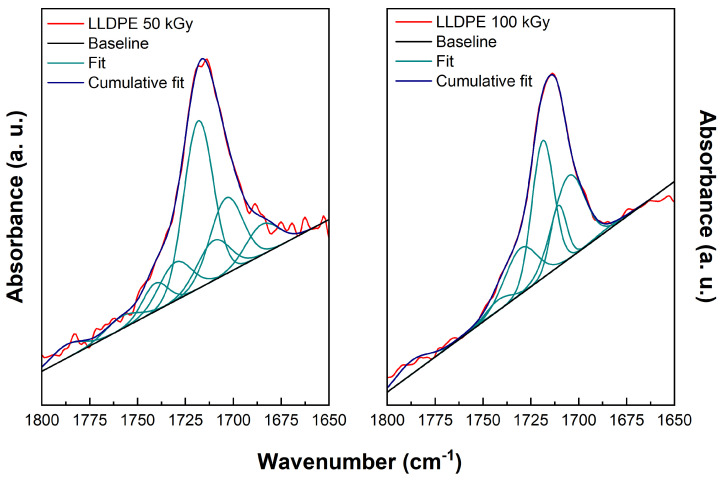
Deconvolution results for LLDPE irradiated at 50 kGy (left) and 100 kGy (right). FWHM of the fitting Gaussians are constrained between 10 and 20 cm${}^{-1}$. The corresponding numerical results are given in Table 3.

**Figure 4:**
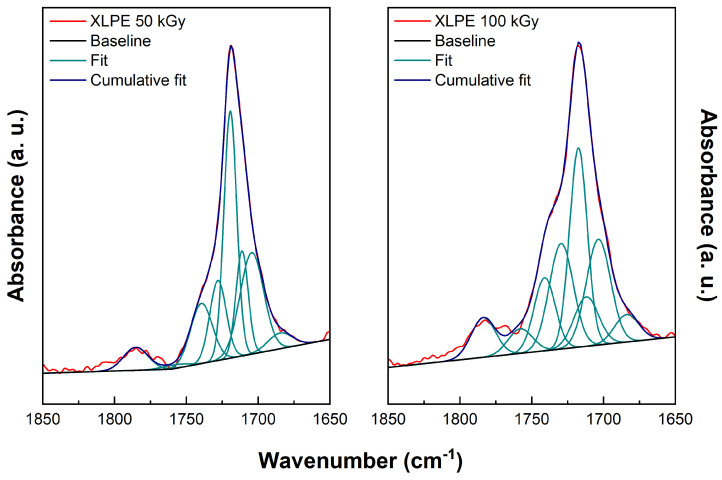
Deconvolution results for XLPE irradiated at 50 kGy (left) and 100 kGy (right). FWHM of the fitting Gaussians are constrained between 10 and 20 cm${}^{-1}$. Associated numerical results are given in Table 4.

Figure 3 presents the deconvolution results obtained for LLDPE irradiated at 50 and 100 kGy; the numerical details are given in Table 3. Analogous results for XLPE irradiated at 50 kGy and 100 kGy are presented in Figure 4 and Table 4.

The main goal of the deconvolutions is to estimate the relative concentrations of the various chemical species produced under irradiation, which can help our understanding of the kinetic mechanisms driving the degradation of polyethylene under irradiation. To extract concentrations from the parameters of the Gaussian function outcome of the fitting procedure, one has to consider that the molar extinction coefficient ϵ of the various species is potentially different. Moreover, as we will see, some questions remain about the identification/assignment of the frequencies and IR intensity associated with the various experimental peaks. For this reason, we first present the theoretical results that we obtained, which can, in some cases, provide missing or alternative data for the analysis of the experimental results.

### 3.2. Calculated IR Spectra

To support the interpretation of the measured FTIR spectra, we calculated using DFPT the infrared spectra of several carbonyl species inserted in our lamellar model at various possible insertion sites. These include crystalline bulk sites and carbonyls at carbon atoms sitting on the lamellar surface or on the chain in the low-density region, mimicking the amorphous fraction of the polymer. We focus here on the carbonyl region of the spectrum. The absolute value of the calculated frequencies is generally a few tens of cm${}^{-1}$ lower than expected frequencies. This probably comes from the approximations used but might also partly depend on the role of the local atomic environment. As a support to the comparison of experimental and theoretical frequencies, we provide in the Supplementary Information further results of calculations for isolated molecules compared to experimental results obtained for gas, liquid phases, or solutions.

However, rather than the absolute value, we focus here on the spread of the frequency for a single species as a function of the position of the carbonyl in the lamellar model. The absolute value of the calculated frequencies is generally on the order of 30 cm${}^{-1}$ lower than the experimental values presented in Table 2. We summarise in Table 5 the average values of calculated frequencies and IR activities, as well as their standard deviation. From the latter, one can extract the FWHM of a corresponding Gaussian distribution, which can be compared with the constraints given in the deconvolution procedure. As an example, the calculated FWHM is ∼22 cm${}^{-1}$ for ketones, ∼23 cm${}^{-1}$ for esters, ∼9 cm${}^{-1}$ for carboxylic acids, and 38 cm${}^{-1}$ for conjugated ketones, although for the latter two species, we have few data.

The discrepancies between theoretical and experimental frequencies are on the order of generally proposed correction factors for quantum chemical calculations of vibrational frequencies [25] for molecules; a scale factor of 1.025 applied to theoretical frequencies would bring them in fair agreement with the experimental reference values. We note that we compared our theoretical carboxylic acid to the free carboxylic acid of Table 2 and not to the H-bonded variant, whose experimentally determined frequency would perfectly match our theoretical value. In this respect, we performed some tests for small molecules, and our theoretical prediction of the vibrational frequency of propionic acid (1772 cm${}^{-1}$) is in fairly good agreement with NIST gas-phase results [43], without the application of the scale factor. This suggests that the role of the local atomic environment and the intermolecular interactions with the characteristic frequencies are yet to be fully understood.

**Figure 5:**
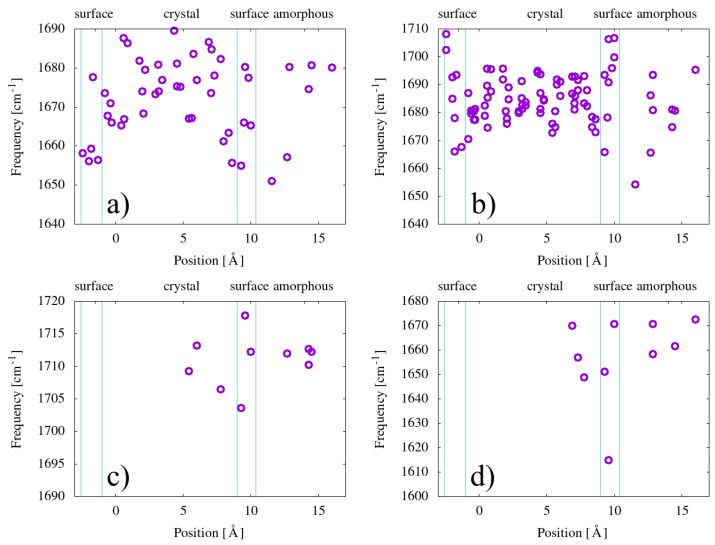
Calculated frequencies of the carbonyl group for (**a**) ketones, (**b**) esters, (**c**) carboxylic acids, and (**d**) enones, presented as a function of the position in the simulation cell in the direction perpendicular to the lamellae. Vertical blue lines delimit regions identified as amorphous, crystalline, and surface, as shown on top of the graph.

We now analyze the theoretical results, both the frequencies and intensities, according to the position of the carbonyl species in the unit cell. To support this goal, we now present in a graphical form (Figure 5) the results already summarised in Table 5, through average values and standard deviations.

**Figure 6:**
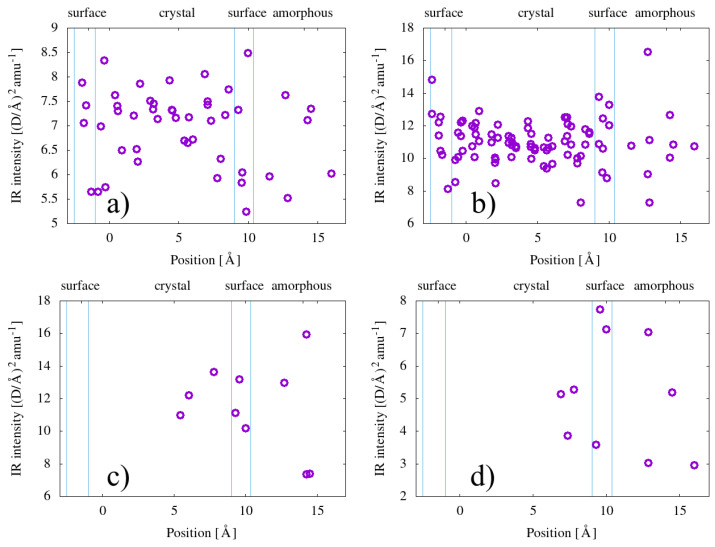
Calculated infrared activities of the carbonyl group for (**a**) ketones, (**b**) esters, (**c**) carboxylic acids, and (**d**) enones, presented as a function of the position in the simulation cell in the direction perpendicular to the lamellae. Vertical blue lines delimit regions identified as amorphous, crystalline, and surface, as shown on top of the graph.

The results, within the limit of our model, do not show a strong difference in dispersions, in terms of both frequencies and IR activities, versus the position. However, in particular for esters, the dispersion in the bulk seems a bit less pronounced than on the surface and in the low-density (amorphous-like) region. Figure 6 also shows that the dispersion of the intensities seems much larger for esters and carboxylic acids than for ketones and enones. We note that we do not find any clear sign of correlation between the frequency and the IR activity of the calculated configurations.

We choose to present the theoretical results for frequencies and IR activities obtained with our lamellar model as a function of the position of the carbonyl in the model structure, in particular as a function of the distance with respect to the lamella surface. The idea is to understand if it could be possible, in principle, to distinguish through the IR signal the position of the carbonyls, even if we know that it is generally admitted that oxygen molecules cannot penetrate and thus induce carbonyl formation inside the crystalline regions. If, however, for some reason, some oxygen happens to be trapped in a crystalline environment or able to penetrate it, our results do not support the idea that inside the crystal the frequency values are less dispersed than in the amorphous region and thus distiguishable in any way. It is true that the lamella in our model is much thinner than real crystalline lamellae in polyethylene, but we stress also the importance of being able to simulate phenomena at the interface between the amorphous and crystal phases, which constitutes a relevant fraction of the material [44,45]; moreover, at lamellae surfaces, at least some of the relevant reactions involved in PE aging and carbonyl production may take place more easily than elsewhere [37].

Let us come now to the dispersion of frequencies as calculated for every single carbonyl type. We note that the Gaussian width allowed in experimental deconvolution procedures (10–20 cm${}^{-1}$) is coherent with what we find from our multiple calculations, at least for ketones and esters, for which we have a sufficiently large set of calculated data. This confirms that the origin of the width of infrared peaks associated with each carbonyl type has to be attributed to the variety of local atomic environments available, although not necessarily due to the amorphous nature of the region where carbonyls are formed: for surface and subsurface regions, we predict similar dispersions.

If we analyze the comparison of theoretical and experimental frequencies of IR active modes, first we note that the theoretical spectrum of our PE model is globally in good agreement with experiments (see Supplementary Information), although with a slight overestimation (1.7%) of the C-H stretching doublet at 2850–2925 cm${}^{-1}$ and a similar underestimation (1.2%) of the bending modes around 1450–1500 cm${}^{-1}$. Concerning the IR active C=O stretching mode used to probe carbonyl species, the underestimation seems to be a bit larger according to Table 5, yet quite satisfactory for DFT calculations of this kind. We performed similar calculations for small molecules containing similar C=O bonds, and we obtained results which are, in some cases, in even better agreement with the experiment (see Supplementary Information). However, we stress that the choice of the experimental reference value is not always straightforward, even for such small molecules, whose frequency can change significantly according to the investigated phase (gas, liquid, solution). Variations might also be expected between PE samples with different densities, additives, and carbonyl concentrations. In order to estimate concentrations, however, we need an even better match with experimental frequencies; thus, we chose to scale the theoretical frequencies with a factor (1.025) that brings them close to experimental results (almost minimizing the standard deviation of the error distribution), while keeping the theoretical ratio between frequencies of different carbonyls.

A last note on the inclusion in the list of carbonyl species of ketone+alcohol pairs, for which we do not have an experimental reference. In a recent paper assessing the viability of various reaction paths through energy barrier calculations [37], we suggest that a relevant ketone production mechanism leads to such pairs. Our calculations of vibrational modes show a slight decrease in the average frequency and, thus, we think it is interesting to include them in the deconvolution procedure.

### 3.3. Concentrations of Carbonyl Species

**Figure 7:**
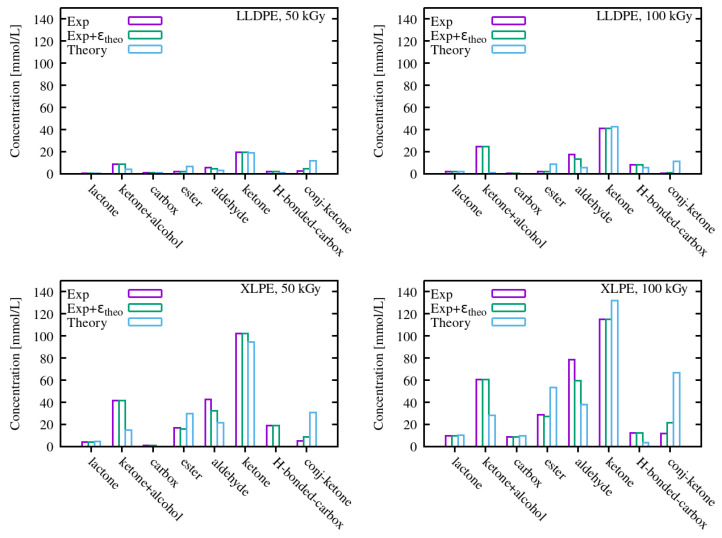
Concentrations of various species as determined from FTIR spectra deconvolution for LLDPE and XLPE for two irradiation doses. We show concentrations obtained with three different sets of fitting parameters, coming only from experiments (“Exp”), only from theory (“Theory”), or from a combination of them (“Exp + $\varepsilon_{theo}$”).

The obtained concentrations are shown in Figure 7. Here we compare three sets of concentrations, the first of which was obtained uniquely from experimental estimations of peak heights and extinction coefficients; the second of which resulted from a combination of experimental peak heights and theoretical IR activities/ϵ; and the third of which was obtained using peak heights from a deconvolution based on theoretical frequencies, combined with theoretical IR activities (except for lactones and H-bonded carboxylic acids, which were not in our calculated set). We note that the main outcomes were qualitatively similar for the three sets of concentrations: ketones were the dominant carbonyl type for both material types and doses, as expected from several previous results on similar materials [22,46,47,48]. Second, the build up of carbonyl species was significantly larger for XLPE than for LLDPE. Third, other species whose concentration is non-negligible were esters, aldehydes, lactones, and conjugated ketones.

For the latter, we note the first clear peculiarity of the concentrations obtained uniquely from theoretical frequencies and IR activities: a much higher concentration of enones (α,β-conjugated ketones), which is probably due to two factors: the relative frequency with respect to other species (closer to ketones in the theoretical frequency set) and the lower relative value of the absorption coefficient in the theoretical set with respect to the experimental one. For the latter, the reference experimental value was obtained for propiophenone, an aromatic compound that is, in fact, different from our enone.

The other main difference is the prevalence of esters over aldehydes in the theoretical analysis, in contrast with the experimental one. This comes not only from the different extinction coefficients, but also from the inversion of the frequency ordering between the two species. The fact that, theoretically, we find a slightly higher frequency for aldehydes than for esters is not excluded by the experimental results presented in ref. [23]. This is not without consequences in assessing the aging mechanisms in polyethylene, because the presence of aldehydes is directly related to the occurrence of β-scission reactions [49], possibly triggered by alkoxy radicals [37]. Esters, on their side, require beforehand the presence of ketone groups that are attacked by alkoxy radicals [50]. As products of secondary reactions, their concentration could be expected to be lower than that of aldehyde groups.

The concentration of ketone+alcohol pairs, which, even in the experimental set of reference data is the only one which is, in fact, coming from our calculations, seems to be in competition with conjugated ketones in the lower frequency section of the carbonyl signal. A larger separation (in frequency) between ketones and ketone+alcohol pairs clearly enhances the calculated concentration of the latter at the expense of conjugated ketones, which represent the lowest frequency of the fitting set. Nevertheless, even in the fully theoretical set, where ketone and ketone–alcohol pairs are very close in frequency (and then in competition in the deconvolution procedure), their concentration is non-negligible, which suggests they cannot be overlooked.

The results, in spite of some discrepancies, show that using the outcome of DFT calculations can be a valuable tool to complement experimental reference data in analyzing experimental spectra of aged polymers and can contribute to a necessary critical assessment [23] of those reference data, especially concerning the absorption coefficients of various species. The theoretical model of PE used in this paper is a rather simplified one, and yet it contains the main ingredients of the complex microstructure of a semicrystalline polymer. This feature is important in order to be able to estimate the influence of the local atomic environment on properties, such as bond stretching frequencies and dipole moment derivatives, which are fairly local in nature but not so much as to guarantee that they can be simulated by isolated molecules in the gas phase.

## 4. Summary and Conclusions

In summary, we have analyzed the carbonyl degradation products in two polyethylene variants (LLDPE and XLPE) using FTIR spectroscopy. Our analysis of experimental infrared spectra, collected on γ-irradiated samples at two doses, combines usual deconvolution procedures and experimental reference data with an extended first principles study of the infrared spectra of various carbonyl species in a model atomic structure.

Our model contains structural features of the crystalline, the amorphous phases, and the interface between them. The theoretical results, in spite of a slight underestimation of the frequency of the carbonyl signals well inside the expected accuracy of the method, provide reliable infrared activities that can complement experimental estimation in the exploitation of experimental spectra. The spread in frequencies according to the atomic site where the carbonyl is inserted gives insight into the influence of the local atomic structure (amorphous, lamella surface, crystal) on the frequencies and IR activities and can be compared to those extracted from deconvolution of experimental spectra.

The estimation of the concentration of carbonyls of various types based on uniquely theoretical data is compared to the outcome of a fully experimental procedure, showing global agreement. Some differences in the ratio of aldehyde to esters and the contribution of conjugated ketones raise the question of the contribution of these species in PE aging under radio-oxidation. Another carbonyl variant that should be further investigated, and which is not systematically included in FTIR analysis of irradiated polyolefins, is a combination of a ketone with an alcohol, as a result of a reaction involving alkoxy radicals.

In conclusion, we think that the approach presented here, combining experimental and theoretical infrared spectra, potentially enhances the ability of FTIR spectroscopy to distinguish and quantify degradation products present in polymers undergoing aging processes. This method can be easily applied to other polymers, provided a representative atomic model is conceived.

## Figures and Tables

**Table 1:** Irradiation conditions.

Nominal Dose (kGy)	Dose Rate (Gy/h)	Real Dose (kGy)
12	1.07	11.96 ± 0.72
24	1.07	24.02 ± 1.44
50	1.06	49.98 ± 3.00
100	1.06	99.98 ± 6.00

**Table 2:** FTIR band assignments in the carbonyl area. We note by $\nu_{exp}^{*}$ the assigned experimental wavenumber (from the reference in the last column) and by $\nu^{min}$ and $\nu^{max}$ the minimum and maximum values allowed in the deconvolution procedure.

FTIR Band Assignment	$\nu_{\exp}^{*}$ [cm${}^{-1}$]	Degree of Freedom		Reference
		$\nu^{\min}$ [cm${}^{-1}$]	$\nu^{\max}$ [cm${}^{-1}$]	
Lactone	1785	1783	1787	Ref. [33]
Ketone + alcohol	1706	1704	1708	Theory, this work
Free carboxylic acid	1756	1754	1758	Refs. [33,34]
Ester	1740	1738	1742	Ref. [35]
Aldehyde	1730	1728	1732	Ref. [35]
Ketone	1718	1716	1720	Ref. [35]
H-bonded carboxylic acid	1710	1708	1712	Ref. [36]
Conjugated ketone	1685	1683	1687	Ref. [32]

**Table 3:** Numerical results of the deconvolution for LLDPE irradiated at 50 kGy (left) and 100 kGy (right). Constraints on peak positions ν are in Table 2. FWHM of the fitting Gaussians are constrained between 10 and 20 cm${}^{-1}$.

FTIR Band Attribution	$\nu_{\exp}^{*}$ [cm${}^{-1}$]	LLDPE 50 kGy			LLDPE 100 kGy		
		ν	Height	FWHM	ν	Height	FWHM
Lactone	1785	1787	1.20 $\times 10^{-4}$	20	1787	3.1${}^{-4}$	20
Ketone + alcohol	1706	1704	6.70 $\times \;10^{-4}$	20	1705	0.00189	19.5
Carboxyl	1756	1758	1.12 $\times \;10^{-4}$	20	1758	≃2.95 $\times \;10^{-5}$	20
Ester	1740	1741	2.47 $\times \;10^{-4}$	15.4	1742	2.45 $\times \;10^{-4}$	15.4
Aldehyde	1730	1730	3.39 $\times \;10^{-4}$	19.7	1730	0.00104	19.6
Ketone	1718	1718	0.00148	18	1719	0.00312	13.5
H-bonded carboxyl	1710	1710	3.52 $\times \;10^{-4}$	20	1711	0.00139	10
Conj. ketone	1685	1685	2.70 $\times \;10^{-4}$	20	1683	6.08 $\times \;10^{-5}$	19.6

**Table 4:** Numerical results of the deconvolution for XLPE irradiated at 50 kGy (left) and 100 kGy (right). Constraints on peak positions ν are in Table 2. FWHM of the fitting Gaussians are constrained between 10 and 20 cm${}^{-1}$.

FTIR Band Attribution	$\nu_{\exp}^{*}$ [cm${}^{-1}$]	XLPE 50 kGy			XLPE 100 kGy		
		ν	Height	FWHM	ν	Height	FWHM
Lactone	1785	1785	7.27 $\times 10^{-4}$	20	1784	0.00175	20
Ketone + alcohol	1706	1705	0.0032	19.4	1704	0.00467	20
Carboxyl	1756	1758	1.21 $\times \;10^{-4}$	20	1758	0.00108	20
Ester	1740	1740	0.0019	17.8	1741	0.00322	17.3
Aldehyde	1730	1728	0.00252	13.5	1730	0.00466	19.7
Ketone	1718	1719	0.00779	10.6	1718	0.00879	14.5
H-bonded carboxyl	1710	1711	0.0033	10.1	1712	0.00218	20
Conjugated ketone	1685	1685	4.95 $\times \;10^{-4}$	20	1684	0.00121	20

**Table 5:** Comparison of theoretical (average) frequencies and IR activities with the corresponding experimental values (frequencies and ϵ are extracted, for each species, from the references mentioned in Table 2). The calculated quantities are averaged over a number of insertion sites of the carbonyl in the lamellar model. The standard deviations of the frequencies and of the IR activities are given in parentheses.

	$\nu_{\mathrm{th}}^{*}$ [cm${}^{-1}$]	$\nu_{\exp}^{*}$ [cm${}^{-1}$]	IR Activity [(D/Å)${}^{2}$amu${}^{-1}$]	ϵ [L mol${}^{-1}$cm${}^{-1}$]
Lactone	–	1785	–	720 [41]
Ester	1684 (9.4)	1740	10.99 (1.422)	450 [42]
Ketone	1672 (9.9)	1718	7.02 (0.827)	300 [42]
Aldehyde	1702 (5.1)	1734	7.25 (1.313)	235 [23]
Free carboxylic acid	1711 (3.7)	1756	11.50 (2.571)	516 [23]
H-bonded carboxyl	–	1710	–	680 [42]
Conjugated ketone	1658 (16.3)	1685	5.10 (1.659)	388 [23]
Ketone + alcohol	1664 (12.1)	–	6.99 (1.291)	–

## Data Availability

The data presented in this study are available on request from the corresponding author.

## Supplementary Materials

The following supporting information can be downloaded at: https://www.mdpi.com/article/10.3390/polym15061537/s1, Figure S1: DART-MS spectrum of pristine LLDPE, Table S1: DART-MS peak attribution, Table S2: FTIR peak attribution, Figure S2: FTIR spectra of pristine PE, Figure S3: XLPE gel fraction, Figure S4: FTIR spectra of irradiated PE, Figures S5–S12 and Tables S3–S10: deconvolution plots and parameters for various constraints on FWHM, Table S11: comparison of R2 of various fits, Figure S13: Theory vs. Experiment for IR spectra of small molecules with carbonyl functions, Figure S14: theoretical spectrum of virgin PE. References [51,52,53] are cited in Supplementary Materials.

## References

[1] Geyer R., Jambeck J.R., Law K.L. (2017). Production, use, and fate of all plastics ever made. Sci. Adv..

[2] Ghatge S., Yang Y., Ahn J.H., Hur H.G. (2020). Biodegradation of polyethylene: A brief review. Appl. Biol. Chem..

[3] Ferry M., Roma G., Cochin F., Esnouf S., Dauvois V., Nizeyimana F., Gervais B., Ngono-Ravache Y., Konings R.J., Stoller R.E. (2020). 3.16—Polymers in the Nuclear Power Industry. Comprehensive Nuclear Materials.

[4] Ferry M., Pellizzi E., Boughattas I., Fromentin E., Dauvois V., de Combarieu G., Coignet P., Cochin F., Ngono-Ravache Y., Balanzat E. (2016). Effect of cumulated dose on hydrogen emission from polyethylene irradiated under oxidative atmosphere using gamma rays and ion beams. Radiat. Phys. Chem..

[5] Seguchi T., Arakawa K., Hayakawa N., Machi S. (1981). Radiation induced oxidative degradation of polymers—IV. Dose rate effects on chemical and mechanical properties. Radiat. Phys. Chem. (1977).

[6] Reynolds A., Bell R., Bryson N., Doyle T., Hall M., Mason L., Quintric L., Terwilliger P. (1995). Dose-rate effects on the radiation-induced oxidation of electric cable used in nuclear power plants. Radiat. Phys. Chem..

[7] Sidi A., Colombani J., Larché J.F., Rivaton A. (2018). Multiscale analysis of the radiooxidative degradation of EVA/EPDM composites. ATH filler and dose rate effect. Radiat. Phys. Chem..

[8] Suraci S.V., Amat S., Hippolyte L., Malechaux A., Fabiani D., Le Gall C., Juan O., Dupuy N. (2022). Ageing evaluation of cable insulations subjected to radiation ageing: Application of principal component analyses to Fourier Transform Infra-Red and dielectric spectroscopy. High Volt..

[9] Fessenden R.W., Schuler R.H. (1963). Electron Spin Resonance Studies of Transient Alkyl Radicals. J. Chem. Phys..

[10] Keyser R.M., Tsuji K., Williams F. (1968). Trapped Electrons in *γ*-Irradiated Polyethylene Indentified by Electron Spin Resonance Spectroscopy. Macromolecules.

[11] Hikmet R., Keller A. (1987). Crystallinity dependent free radical formation and decay in irradiated polyethylene in the presence of oxygen. Int. J. Radiat. Appl. Instrum. Part C Radiat. Phys. Chem..

[12] Salih M., Buttafava A., Ravasio U., Mariani M., Faucitano A. (2007). EPR investigation on radiation-induced graft copolymerization of styrene onto polyethylene: Energy transfer effects. Radiat. Phys. Chem..

[13] Przybytniak G., Sadło J., Walo M., Wróbel N., Žák P. (2020). Comparison of radical processes in non-aged and radiation-aged polyethylene unprotected or protected by antioxidants. Mater. Today Commun..

[14] Osawa Z., Kuroda H. (1986). Differences in polyene formation between polyethylene and polypropylene during photo-irradiation. Polymer Photochem..

[15] Teyssedre G., Cissé L., Laurent C., Massines F., Tiemblo P. (1998). Spectral Analysis of Optical Emission Due to Isothermal Charge Recombination in Polyolefins. IEEE Trans. Dielectr. Electr. Insul..

[16] Randall J., Zoepfl F., Silverman J. (1983). High-resolution solution carbon 13 NMR measurements of irradiated polyethylene. Radiat. Phys. Chem. (1977).

[17] Palmas P., Le Campion L., Bourgeoisat C., Martel L. (2001). Curing and thermal ageing of elastomers as studied by 1H broadband and 13C high-resolution solid-state NMR. Polymer.

[18] Fallgatter M.B., Dole M. (1964). The Radiation Chemistry of Polyethylene. VII. Polyene Formation1. J. Phys. Chem..

[19] Mailhot B., Gardette J.L. (1992). Polystyrene photooxidation. 2. A pseudo wavelength effect. Macromolecules.

[20] Möller K., Gevert T. (1996). A solid-state investigation of the desorption/evaporation of hindered phenols from low density polyethylene using FTIR and UV spectroscopy with integrating sphere: The effect of molecular size on the desorption. J. Appl. Polym. Sci..

[21] Rivaton A., Cambon S., Gardette J.L. (2005). Radiochemical ageing of EPDM elastomers: 2. Identification and quantification of chemical changes in EPDM and EPR films *γ*-irradiated under oxygen atmosphere. Nucl. Instrum. Methods Phys. Res. Sect. B Beam Interact. Mater. Atoms.

[22] Gardette M., Perthue A., Gardette J.L., Janecska T., Földes E., Pukánszky B., Therias S. (2013). Photo- and thermal-oxidation of polyethylene: Comparison of mechanisms and influence of unsaturation content. Polym. Degrad. Stab..

[23] Celina M.C., Linde E., Martinez E. (2021). Carbonyl Identification and Quantification Uncertainties for Oxidative Polymer Degradation. Polym. Degrad. Stab..

[24] Baroni S., de Gironcoli S., Dal Corso A., Giannozzi P. (2001). Phonons and related crystal properties from density-functional perturbation theory. Rev. Mod. Phys..

[25] Schott A.P., Radom L. (1996). Harmonic Vibrational Frequencies: An Evaluation of Hartree-Fock, Moller-Plesset, Quadratic Configuration Interaction, Density Functional Theory, and Semiempirical ScaleFactors. J. Phys. Chem..

[26] Porezag D., Pederson M.R. (1996). Infrared intensities and Raman-scattering activities within density-functional theory. Phys. Rev. B.

[27] Halls M.D., Schlegel H.B. (1998). Comparison of the performance of local, gradient-corrected, and hybrid density functional models in predicting infrared intensities. J. Chem. Phys..

[28] Gaigeot M.P., Sprik M. (2003). Ab Initio Molecular Dynamics Computation of the Infrared Spectrum of Aqueous Uracil. J. Phys. Chem. B.

[29] Welch M.D., Montgomery W., Balan E., Lerch P. (2012). Insights into the high-pressure behavior of kaolinite from infrared spectroscopy and quantum-mechanical calculations. Phys. Chem. Miner..

[30] Ferry M., Carpentier F., Cornaton M. (2021). Radio-Oxidation Ageing of XLPE Containing Different Additives and Filler: Effect on the Gases Emission and Consumption. Polymers.

[31] Gillen K.T., Clough R.L. (1991). Quantitative Confirmation of Simple Theoretical Models for Diffusion-Limited Oxidation. Radiation Effects on Polymers.

[32] Ngono-Ravache Y., Damaj Z., Dannoux-Papin A., Ferry M., Esnouf S., Cochin F., De Combarieu G., Balanzat E. (2015). Effect of swift heavy ions on an EPDM elastomer in the presence of oxygen: LET effect on the radiation-induced chemical ageing. Polym. Degrad. Stab..

[33] Boullier I. (2000). Étude du comportement de polyoléfines et de polymères fluorés dans des conditions de vieillissement naturel et Accéléré. Ph.D. Thesis.

[34] Lacoste J., Carlsson D.J. (1992). Gamma-, photo-, and thermally-initiated oxidation of linear low density polyethylene: A quantitative comparison of oxidation products. J. Polym. Sci. Part A Polym. Chem..

[35] Luongo J.P. (1960). Infrared study of oxygenated groups formed in polyethylene during oxidation. J. Polym. Sci..

[36] Teissèdre G., Pilichowski J., Lacoste J. (1994). Photoageing of polyolefins. I. Photolysis of low molecular weight hydroperoxide at *λ*≥ 300 nm. Polym. Degrad. Stab..

[37] Ahn Y., Roma G., Colin X. (2022). Elucidating the Role of Alkoxy Radicals in Polyethylene Radio-Oxidation Kinetics. Macromolecules.

[38] Klimeš J., Bowler D.R., Michaelides A. (2011). Van der Waals density functionals applied to solids. Phys. Rev. B.

[39] Roma G., Bruneval F., Martin-Samos L. (2018). Optical Properties of Saturated and Unsaturated Carbonyl Defects in Polyethylene. J. Phys. Chem. B.

[40] Giannozzi P., Baroni S., Bonini N., Calandra M., Car R., Cavazzoni C., Ceresoli D., Chiarotti G.L., Cococcioni M., Dabo I. (2009). QUANTUM ESPRESSO: A modular and open-source software project for quantum simulations of materials. J. Phys. Condens. Matter.

[41] Lacoste J., Carlsson D., Falicki S., Wiles D. (1991). Polyethylene hydroperoxide decomposition products. Polym. Degrad. Stab..

[42] Carlsson D.J., Wiles D.M. (1969). The Photodegradation of Polypropylene Films. II. Photolysis of Ketonic Oxidation Products. Macromolecules.

[43] Wallace W.E., Sadtler Research Labs Under US-EPA Contract (2018). NIST Standard Reference Database 35, NIST/EPA Gas-Phase Infrared Database JCAMP Format.

[44] Zhou H., Wilkes G. (1997). Comparison of lamellar thickness and its distribution determined from d.s.c., SAXS, TEM and AFM for high-density polyethylene films having a stacked lamellar morphology. Polymer.

[45] Savage R.C., Mullin N., Hobbs J.K. (2015). Molecular Conformation at the Crystal–Amorphous Interface in Polyethylene. Macromolecules.

[46] Fodor Z., Iring M., Tüdős F., Kelen T. (1984). Determination of carbonyl-containing functional groups in oxidized polyethylene. J. Polym. Sci. Polym. Chem. Ed..

[47] Costa L., Luda M., Trossarelli L. (1997). Ultra high molecular weight polyethylene—II. Thermal-and photo-oxidation. Polym. Degrad. Stab..

[48] Salvalaggio M., Bagatin R., Fornaroli M., Fanutti S., Palmery S., Battistel E. (2006). Multi-component analysis of low-density polyethylene oxidative degradation. Polym. Degrad. Stab..

[49] Khelidj N., Colin X., Audouin L., Verdu J., Monchy-Leroy C., Prunier V. (2006). Oxidation of polyethylene under irradiation at low temperature and low dose rate. Part I. The case of “pure” radiochemical initiation. Polym. Degrad. Stab..

[50] Cambon S. (2001). Étude du mécanisme de dégradation radiochimique d’un élastomère de type EPDM. Ph.D. Thesis.

[51] Carlsson D.J., Brousseau R., Zhang C., Wiles D.M. (1988). Identification of Products from Polyolefin Oxidation by Derivatization Reactions. Chemical Reactions on Polymers.

[52] Hulse G.E., Kersting R.J., Warfel D.R. (1981). Chemistry of dicumyl peroxide-induced crosslinking of linear polyethylene. J. Polym. Sci. Polym. Chem. Ed..

[53] Xu A., Roland S., Colin X. (2020). Physico-chemical characterization of the blooming of Irganox 1076® antioxidant onto the surface of a silane-crosslinked polyethylene. Polym. Degrad. Stab..

