# Supplementary material for: Combining Experimental and Theoretical Tools to Probe Radio-Oxidation Products in Polyethylene
Source: Polymers (Basel). 2023 Mar 20;15(6):1537. doi: 10.3390/polym15061537 (PMC10057136; doi:10.3390/polym15061537)
Supplement: Supplementary file 1 [file polymers-15-01537-s001.zip › polymers-2236960-supplementary.pdf]

Supplementary Information to “Combining experimental and theoretical tools to probe radio-oxidation products in polyethylene”

Muriel Ferry[1], Yunho Ahn[2], Florian Le Dantec[1], Yvette Ngono[3] and Guido Roma[2]

[1] *Université Paris-Saclay, CEA, Service de Physico-Chimie (SPC), F-91191 Gif-sur-Yvette, France.*

[2] *Université Paris-Saclay, CEA, Service de Recherches en Corrosion et Comportement des Matériaux (SRMP), F-91191 Gif-sur-Yvette, France.*

[3] *CIMAP, CEA-CNRS-ENSICAEN-UNICAEN, Normandie Université, F-14050 Caen Cedex 04, France.*

1. Experimental characterization of the materials

1.1. Methods

1.1.1. Gel fraction determination

Mass m_0 of about 10-20 mg of polymer is dissolved in 60 mL of p-xylene and heated at 190° C for 5 h under constant reflux. Non-solubilized polymer is recovered and its mass m_1 is dried under vacuum at 80° C. overnight. After drying, the mass m_2 of the polymer is measured. Gel fraction $\%_{gel}$ is determined using Equation S1 while swelling factor f is calculated using Equation S2.

$$\%_{gel} = \frac{m_1}{m_0} \cdot 100 \qquad \text{Equation S1}$$

$$f = \frac{m_1}{m_2} \cdot 100 \qquad \text{Equation S2}$$

1.1.2. Direct Analysis in Real Time Mass Spectrometry (DART-MS)

A DART SVP direct analysis in real time (DART) ion source (Ion-Sense, Inc., Saugus, MA, USA) was interfaced with a LCT XE Premier (Waters, Manchester, UK) for sample analysis. The DART was operated in the negative ion mode at 500°C with ultrapure helium and nitrogen (purities at 99.999%) as carrier gas at 5.5 bars. The grid electrode voltage was set to 3500 V.

The LCT XE Premier mass spectrometer settings were 500°C for the source temperature and 30 V for the sample cone voltage. Spectra are acquired at 1 scan per second over a mass range of m/z 50 - 5000. All data analysis was accomplished using MassLynx Software (Waters, Manchester, UK).

1.2. Initial characterization

1.2.1. Gel fraction

Two polymers were studied in this work: LLDPE and XLPE. Gel fraction of both materials were measured prior to irradiation and are respectively equal to 0 % and 67 ± 1 %.

1.2.2. DART-MS analyses

LLDPE purity was evaluated by DART-MS in negative mode. In such mode, additives can be observed without any contribution of the polymeric material - polyethylene does not present heteroatoms allowing anion formation in its structure. Figure S1 shows four peaks which can all be attributed to Irganox 1076 (see Table S1).

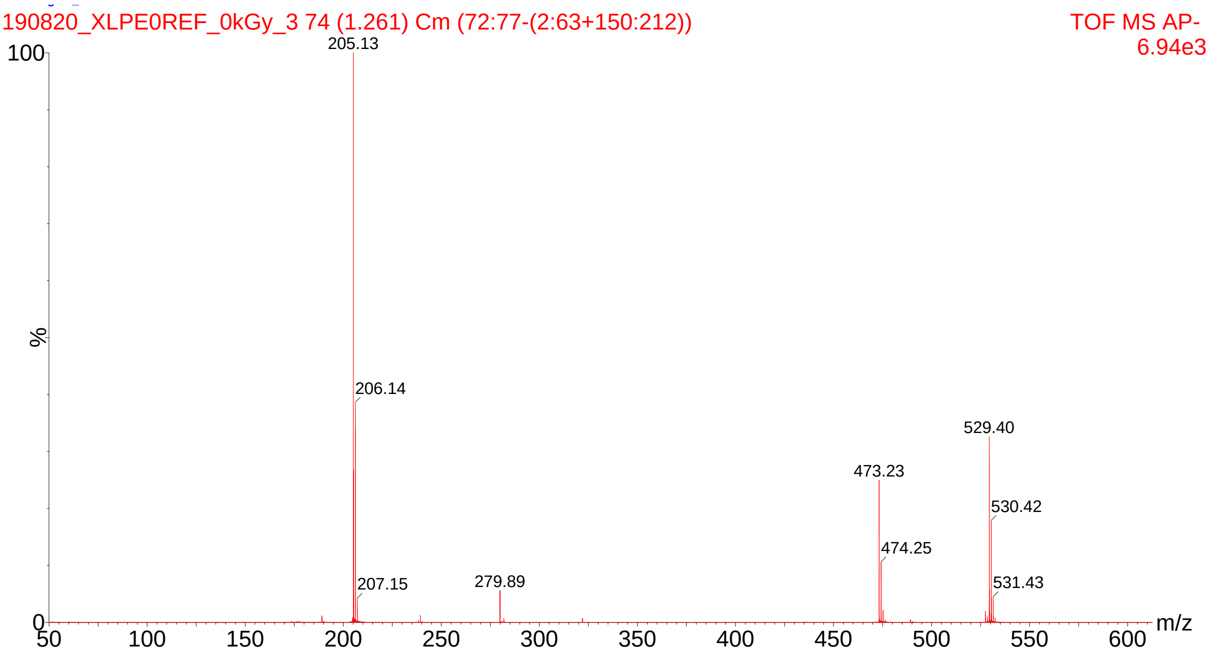

Figure S1. DART-MS spectrum of pristine LLDPE.

m/z	Peak attribution	m/z	Peak attribution
529	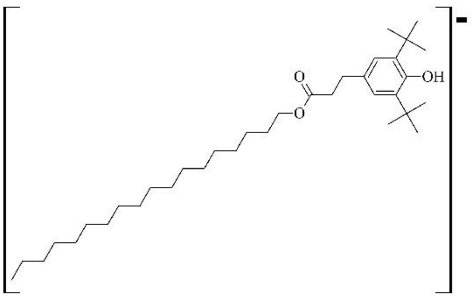	280	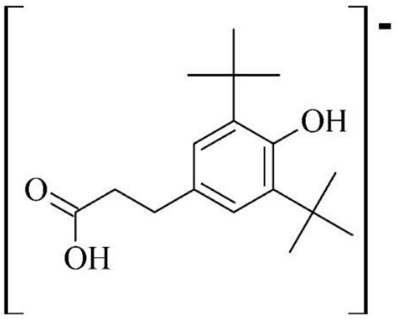
473	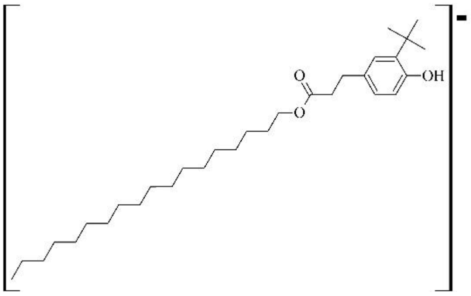	205	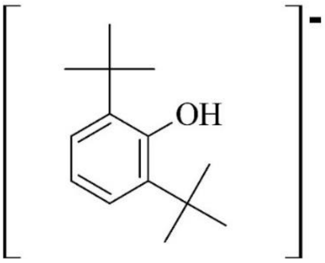

Table S1. Attribution of peaks in pristine LLDPE obtained by DART-MS in negative mode at 500°C.

1.2.3. FTIR spectroscopy

It has been evidenced by DART-MS that Irganox 1076 is present in pristine LLDPE. In the case of XLPE, DCP has been added to allow the crosslinking reactions to happen; hence, some decomposition products of this molecule are also expected in the pristine XLPE material. FTIR characteristic bands of polyethylene, of Irganox 1076 and of DCP decomposition products are given in Table S2.

It can be evidenced on the FTIR spectra of LLDPE and XLPE, given in Figure S2, that in addition to the polyethylene characteristic bands, both spectra show characteristic bands of Irganox 1076. XLPE FTIR spectrum might also present bands coming from the decomposition products of DCP, but if so then they are very weak.

Polyethylene	Wavenumber (cm^{-1}) Irganox 1076 (Xu et al., 2020)	DCP decomposition products (Hulse et al., 1981)	FTIR band attribution
	3639		$\nu(-OH)$ – phenol
		3448	$\nu(-OH)$ – phenol
	3002		$\nu(CH)$ – aromatic ring
	2953		$\nu(CH)$ – CH_3
2925			$\nu_a(CH_2)$
2853			$\nu_s(CH_2)$
2018			1301 + 720 harmonic
	1733		$\nu(C=O)$ – ester
		1724	$\nu(C=O)$ – ketone
		1694	$\nu(C=O)$ – aryl ketone
1470			$\delta(CH_2)$ – crystalline phase
1460			$\delta(CH_2)$
1456			$\delta(CH_2)$ – amorphous phase
1369			$\omega(CH_2)$
1353			$\omega(CH_2)$ – amorphous phase
1301			$\omega(CH_2)$ – amorphous phase
1080			$\nu(CC)$ – amorphous phase
1065			$\nu(CC)$
	1400 – 1000		$\nu(C-O)$ – ester and phenol
		952	Conjugated unsaturation*
	870		$\delta(CH)$ – aromatic ring
		843	Trisubstituted ethylene
		829	Trisubstituted ethylene
		760	Trisubstituted ethylene
730			$R(CH_2)$ – crystalline phase
725			$R(CH_2)$ – amorphous phase
720			$R(CH_2)$ – crystalline phase
		699	$\delta(CH)$ – aromatic ring
		689	$\delta(CH)$ – aromatic ring

Table S2. Attribution and relative intensity of FTIR bands of polyethylene. ν: stretching, δ: bending, ω: wagging, R: rocking. Subscripts s: symmetric and a: antisymmetric. *: uncertain attribution.

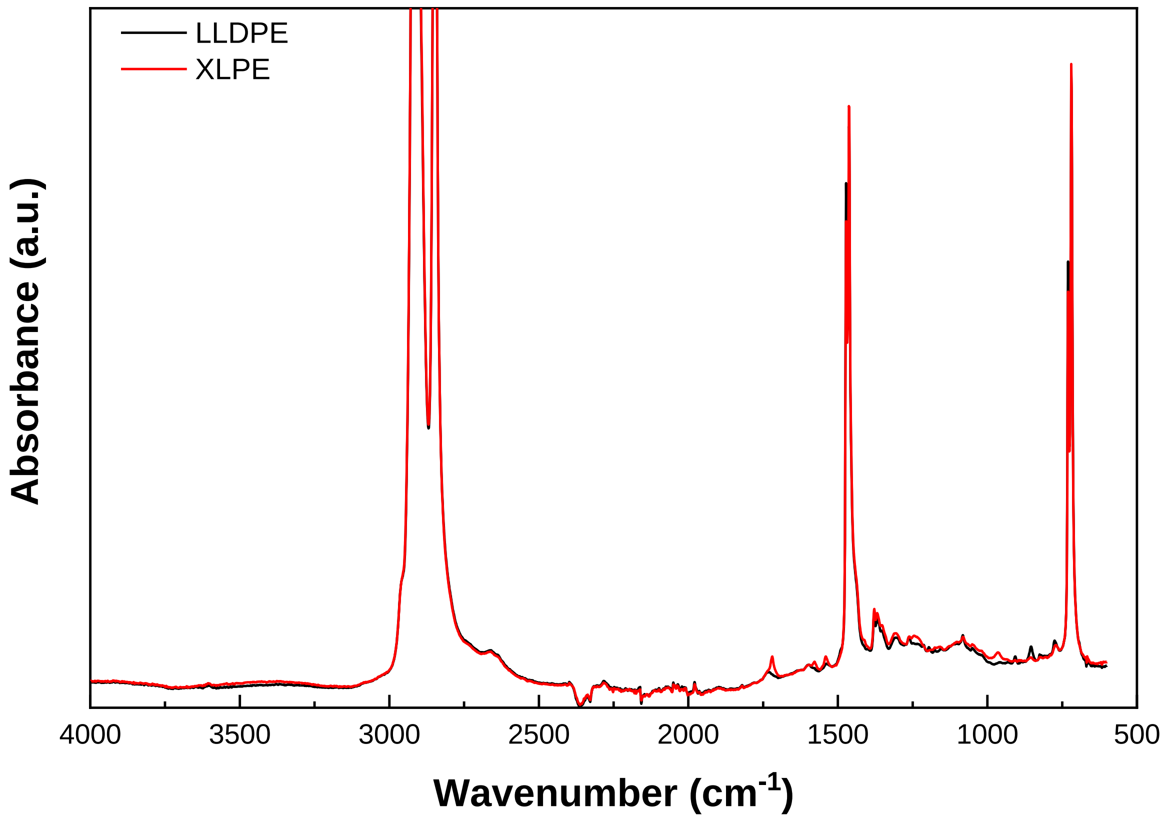

Figure S2. FTIR spectra of LLDPE and of XLPE.

1.3. Characterization after radio-oxidation

1.3.1. Gel fraction quantification

Gel fraction of LLDPE was null prior to irradiation and remains null up to 100 kGy. Figure S3 presents the evolution of this parameter a function of dose in the case of XLPE. This plot evidences a decrease of this parameter with dose, which implies that chain scission is predominant over crosslinking when XLPE is radio-oxidized. Moreover, the evolution is linear with dose: saturation is not attained at 100 kGy.

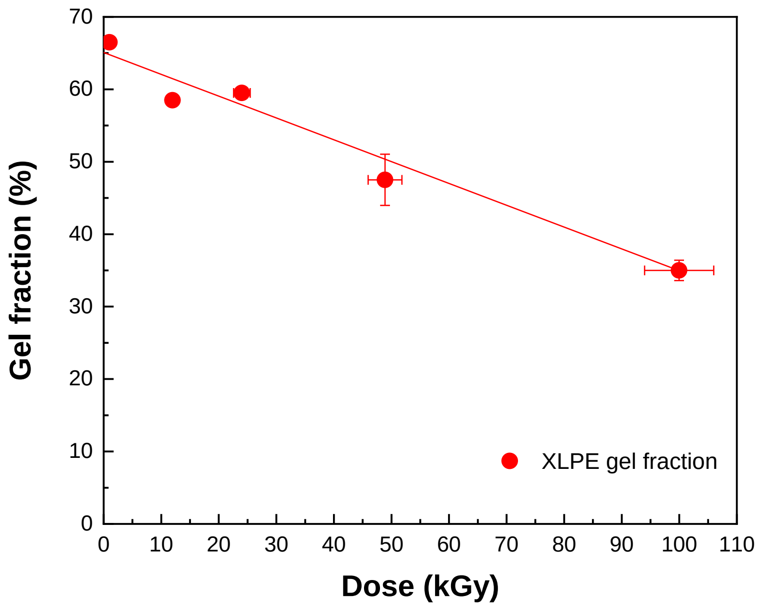

Figure S3. XLPE Gel fraction evolution as a function of dose.

1.3.2. FTIR spectroscopy

FTIR spectra of LLDPE and of XLPE are given at the different doses in Figure S4, top and bottom respectively. It can be observed that, as usual in case of radio-oxidized aliphatic polymers, there is an increase in the 3600-3200 cm^{-1} area attributable to the $\nu(-OH)$ vibration and in the 1800-1650 cm^{-1} area relative to the $\nu(C=O)$ vibration. A less usual intense peak, centred in both materials around 1240 cm^{-1}, is observed: it is tentatively attributed to $\nu(C-O)$ vibration.

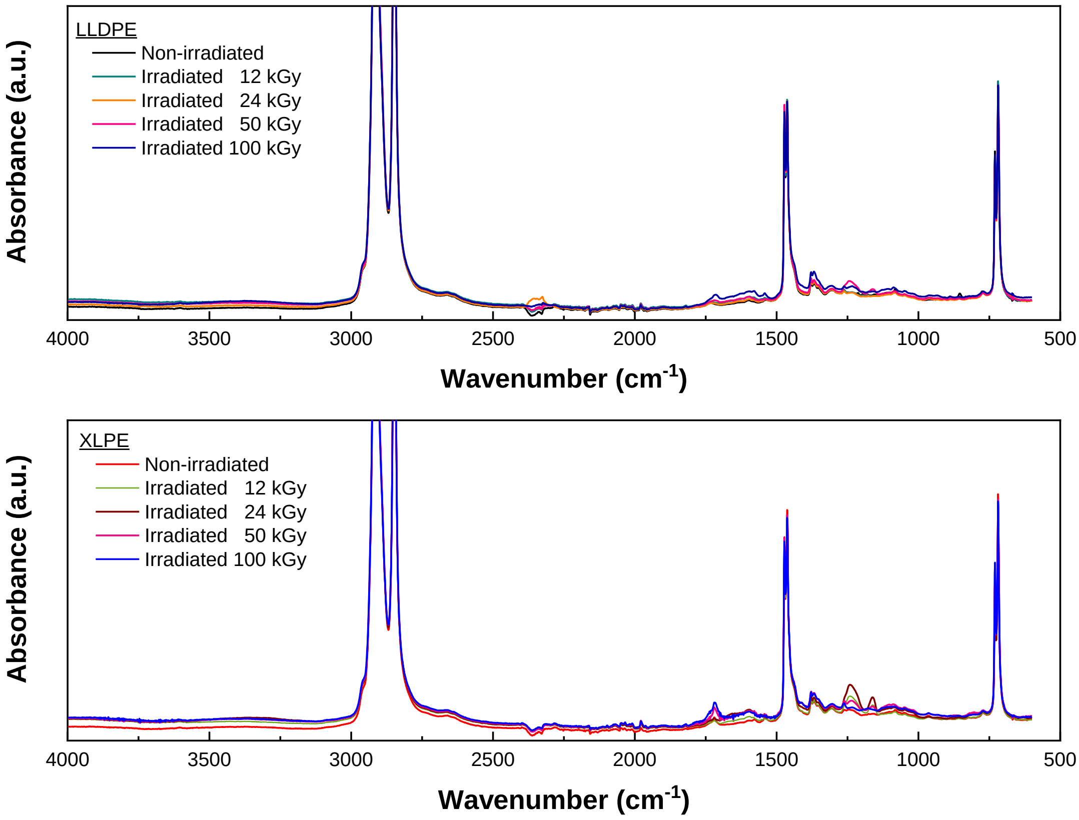

Figure S4. FTIR spectra of LLDPE (top) and of XLPE (bottom) at the different doses of this study.

2. Deconvolution results

In this section we present further deconvolution results that are not in the main body of the article. We note that, initially, we performed deconvolutions including a band at 1770 cm^{-1}, attributed to peresters (Carlsson et al., 1988). For the sake of consistency, and given the fact that a value of the extinction coefficient ε could not be found for peresters, we finally discarded it from all the deconvolutions. R^2 values remain very good.

2.1. Width at half maximum chosen comprised between 5 and 15 cm^{-1}

Figure S5 presents the deconvolution results obtained for LLDPE irradiated at 50 kGy (left) and 100 kGy (right), peak position parameters being given in the main body of the article and full width at half maximum (FWHM) chosen between 5 and 15 cm^{-1}. Numerical results are given in Table S3.

An equivalent presentation for XLPE irradiated at 50 kGy and 100 kGy is given in Figure S6 and Table S4.

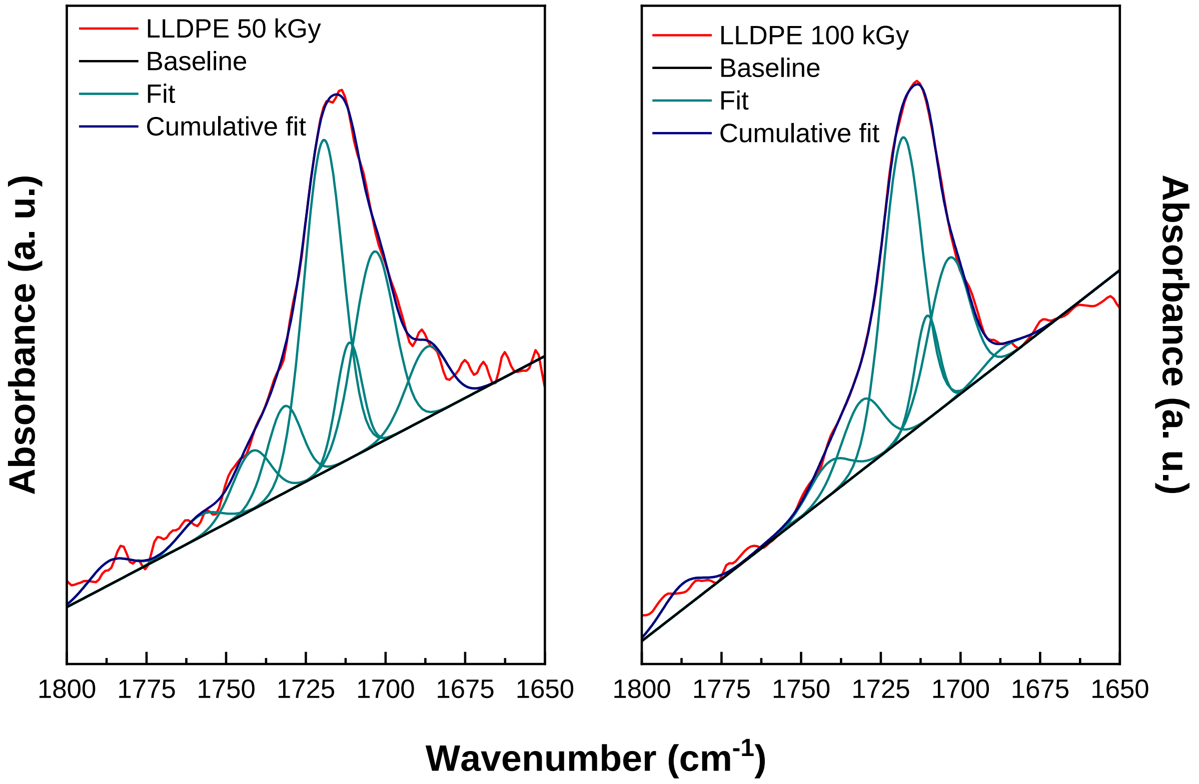

Figure S5. Deconvolution results for LLDPE irradiated at 50 kGy (left) and 100 kGy (right). Peak position parameters are given in the main body of the article. FWHM were constrained between 5 and 15 cm^{-1}. Associated numerical results are given in Table S3.

FTIR band attribution	Wavenumber (cm^{-1})	LLDPE 50 kGy			LLDPE 100 kGy		
		Position	Height	FWHM	Position	Height	FWHM
Lactone	1785.0	1786.7	$1.28\ 10^{-4}$	15	1787.0	$3.32\ 10^{-4}$	15
Ketone + alcohol	1705.6	1703.6	0.00103	15	1703.6	0.00187	15
Free carboxylic acid of peracid	1756.0	1758.0	$1.17\ 10^{-4}$	15	1758.0	$3.00\ 10^{-5}$	15
Ester	1740.0	1742.0	$3.13\ 10^{-4}$	14.0	1741.3	$4.37\ 10^{-4}$	15
Aldehyde	1730.0	1731.7	$4.60\ 10^{-4}$	12.7	1730.9	$9.04\ 10^{-4}$	15
Ketone	1718.0	1719.5	0.00177	14.4	1718.2	0.00389	14.2
Carboxylic acid	1710.0	1711.4	$6.17\ 10^{-4}$	9.3	1710.6	0.00134	8.7
Conjugated ketone	1685.0	1687.0	$3.78\ 10^{-4}$	15	1687.0	$1.70\ 10^{-4}$	15

Table S3. Numerical results of deconvolution for LLDPE irradiated at 50 kGy (left) and 100 kGy (right). Peak position parameters are given in the main body of the article. FWHM between were constrained between 5 and 15 cm^{-1}.

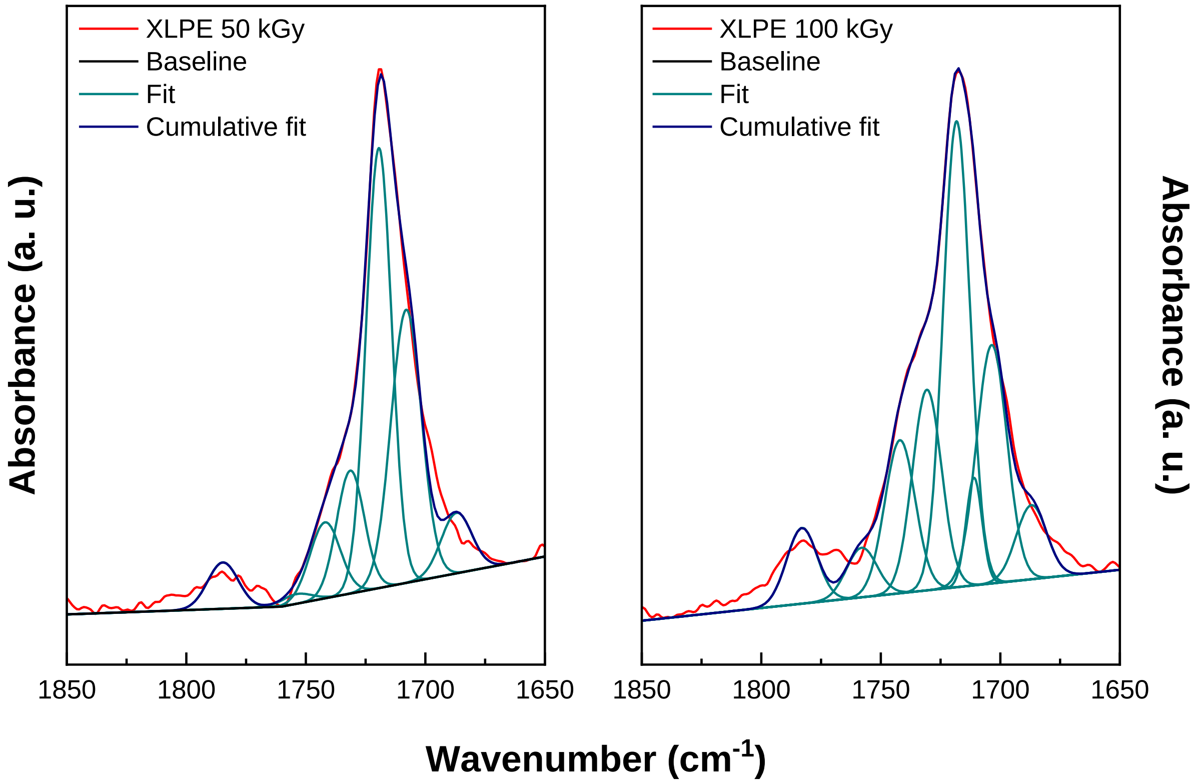

Figure S6. Deconvolution results for XLPE irradiated at 50 kGy (left) and 100 kGy (right). Peak position parameters are given in the main body of the article. FWHM were constrained between 5 and 15 cm^{-1}. Associated numerical results are given in Table S4.

FTIR band attribution	Wavenumber (cm^{-1})	XLPE 50 kGy			LLDDPE 100 kGy		
		Position	Height	FWHM	Position	Height	FWHM
Lactone	1785.0	1784.7	$8.81\ 10^{-4}$	15	1783.0	0.00195	15
Ketone + alcohol	1705.6	1707.6	$\simeq 0$	10	1703.6	0.00615	15
Free carboxylic acid of peracid	1756.0	1754.0	$1.85\ 10^{-4}$	15	1758.0	0.00128	15
Ester	1740.0	1742.0	0.00144	15	1742.0	0.00395	15
Aldehyde	1730.0	1731.4	0.00233	13.6	1730.7	0.00518	14.3
Ketone	1718.0	1719.4	0.00835	12.5	1718.3	0.01203	13.1
Carboxylic acid	1710.0	1708.0	0.00518	15	1711.0	0.00278	7.9
Conjugated ketone	1685.0	1687.0	0.00115	15	1687.0	0.00191	15

Table S4. Numerical results of deconvolution for XLPE irradiated at 50 kGy (left) and 100 kGy (right). Peak position parameters are given in the main body of the article. FWHM were constrained between 5 and 15 cm^{-1}.

2.2. FWHM comprised between 15 and 25 cm^{-1}

Figure S7 presents the deconvolution results obtained for LLDPE irradiated at 50 kGy (left) and 100 kGy (right), peak position parameters being given in the main body of the article and FWHM between 15 and 25 cm^{-1}. Numerical results are given in Table S5.

An equivalent presentation for XLPE irradiated at 50 kGy and 100 kGy is given in Figure S8 and Table S6.

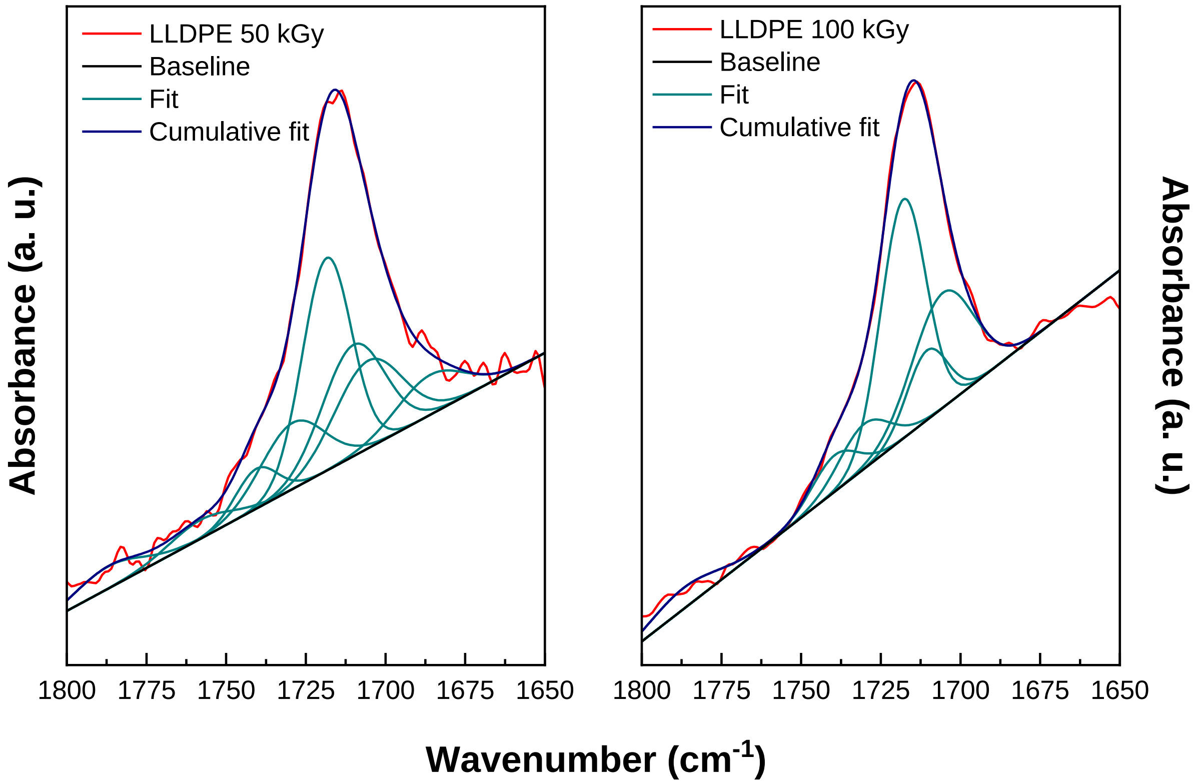

Figure S7. Deconvolution results for LLDPE irradiated at 50 kGy (left) and 100 kGy (right). Peak position parameters are given in the main body of the article. FWHM were constrained between 15 and 25 cm^{-1}. Associated numerical results are given in Table S5.

FTIR band attribution	Wavenumber (cm^{-1})	LLDPE 50 kGy			LLDPE 100 kGy		
		Position	Height	FWHM	Position	Height	FWHM
Lactone	1785.0	1787.0	$1.18\ 10^{-4}$	25	1787.0	$2.71\ 10^{-4}$	25
Ketone + alcohol	1705.6	1705.5	$4.66\ 10^{-4}$	25	1705.5	0.00148	22.5
Free carboxylic acid of peracid	1756.0	1758.0	$9.98\ 10^{-5}$	23.1	1755.4	≃ 0	19.4
Ester	1740.0	1740.5	$2.12\ 10^{-4}$	15.4	1739.4	$4.71\ 10^{-4}$	16.7
Aldehyde	1730.0	1729.1	$3.52\ 10^{-4}$	23.5	1730.2	$5.79\ 10^{-4}$	18.4
Ketone	1718.0	1718.5	0.00113	18.1	1717.9	0.00308	16.7
Carboxylic acid	1710.0	1710.1	$5.92\ 10^{-4}$	22.9	1710.5	$8.99\ 10^{-4}$	15
Conjugated ketone	1685.0	1686.0	$2.12\ 10^{-4}$	25	1683.0	0	24.9

Table S5. Numerical results of deconvolution for LLDPE irradiated at 50 kGy (left) and 100 kGy (right). Peak position parameters are given in the main body of the article. FWHM were constrained between 15 and 25 cm^{-1}.

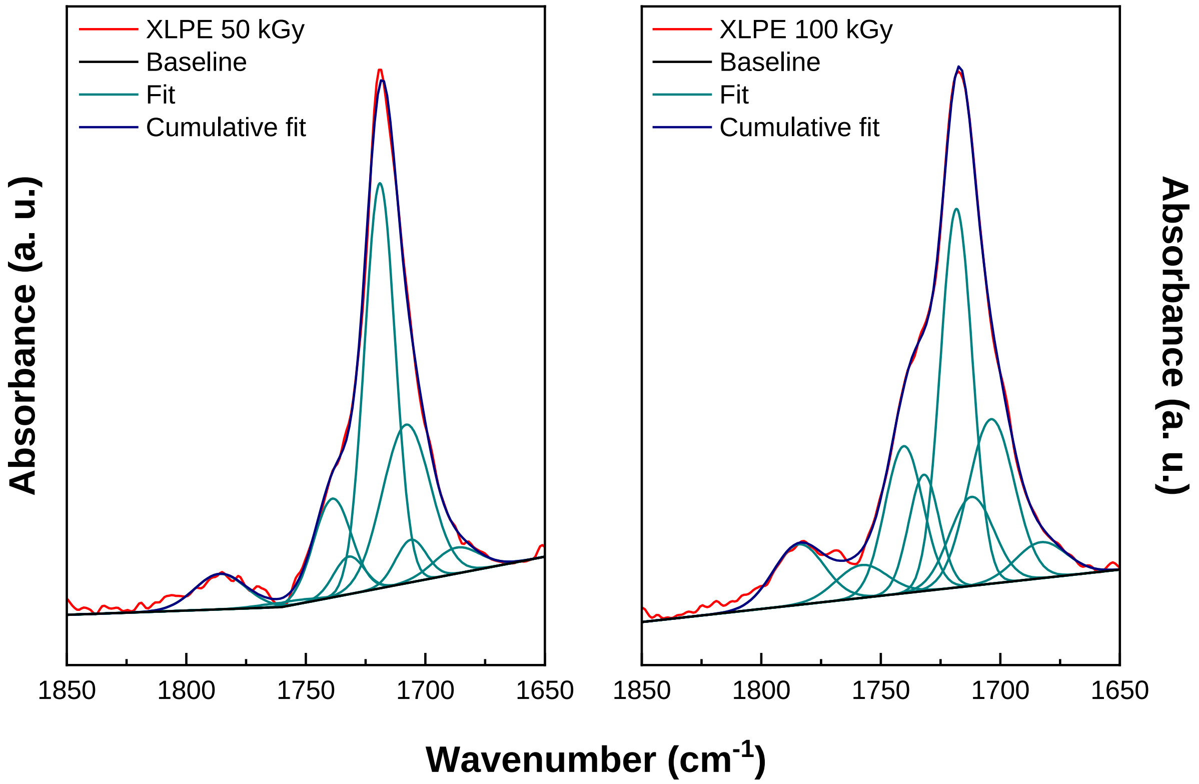

Figure S8. Deconvolution results for XLPE irradiated at 50 kGy (left) and 100 kGy (right). Peak position parameters are given in the main body of the article. FWHM were constrained between 15 and 25 cm^{-1}. Associated numerical results are given in Table S6.

**FTIR band attribution**	**Wavenumber (cm^{-1})**	**XLPE 50 kGy**			**XLPE 100 kGy**		
		**Position**	**Height**	**FWHM**	**Position**	**Height**	**FWHM**
Lactone	1785.0	1785.9	$6.72\ 10^{-4}$	25	1784.3	0.00156	25
Ketone + alcohol	1705.6	1706.0	$8.09\ 10^{-4}$	15	1703.8	0.00425	22.6
Free carboxylic acid of peracid	1756.0	1758.0	$8.35\ 10^{-5}$	25	1758.0	$8.49\ 10^{-4}$	25
Ester	1740.0	1738.9	0.00187	18.5	1740.3	0.00380	18.7
Aldehyde	1730.0	1732.0	$7.14\ 10^{-4}$	15	1732.0	0.00300	15
Ketone	1718.0	1718.0	0.00768	15	1718.4	0.00977	15.7
Carboxylic acid	1710.0	1708.0	0.00301	23.4	1712.0	0.00230	21.8
Conjugated ketone	1685.0	1687.0	$4.93\ 10^{-4}$	22.5	1683.0	$9.3\ 10^{-4}$	25

Table S6. Numerical results of deconvolution for XLPE irradiated at 50 kGy (left) and 100 kGy (right). Peak position parameters are given in the main body of the article. FWHM were constrained between 15 and 25 cm^{-1}.

2.3. FWHM comprised between 20 and 30 cm^{-1}

Figure S9 presents the deconvolution results obtained for LLDPE irradiated at 50 kGy (left) and 100 kGy (right), peak position parameters being given in the main body of the article and FWHM between 20 and 30 cm^{-1}. Numerical results are given in Table S7.

An equivalent presentation for XLPE irradiated at 50 kGy and 100 kGy is given in Figure S10 and Table S8.

Within these specifications, there were difficulties to obtain a fit convergence because of mutual dependencies between parameters. In the case of XLPE 50 kGy, no convergence could be obtained and the adjustment stopped after 1200 iterations. No R-square is thus given in the Comparison section and data are added in italic in Table S8.

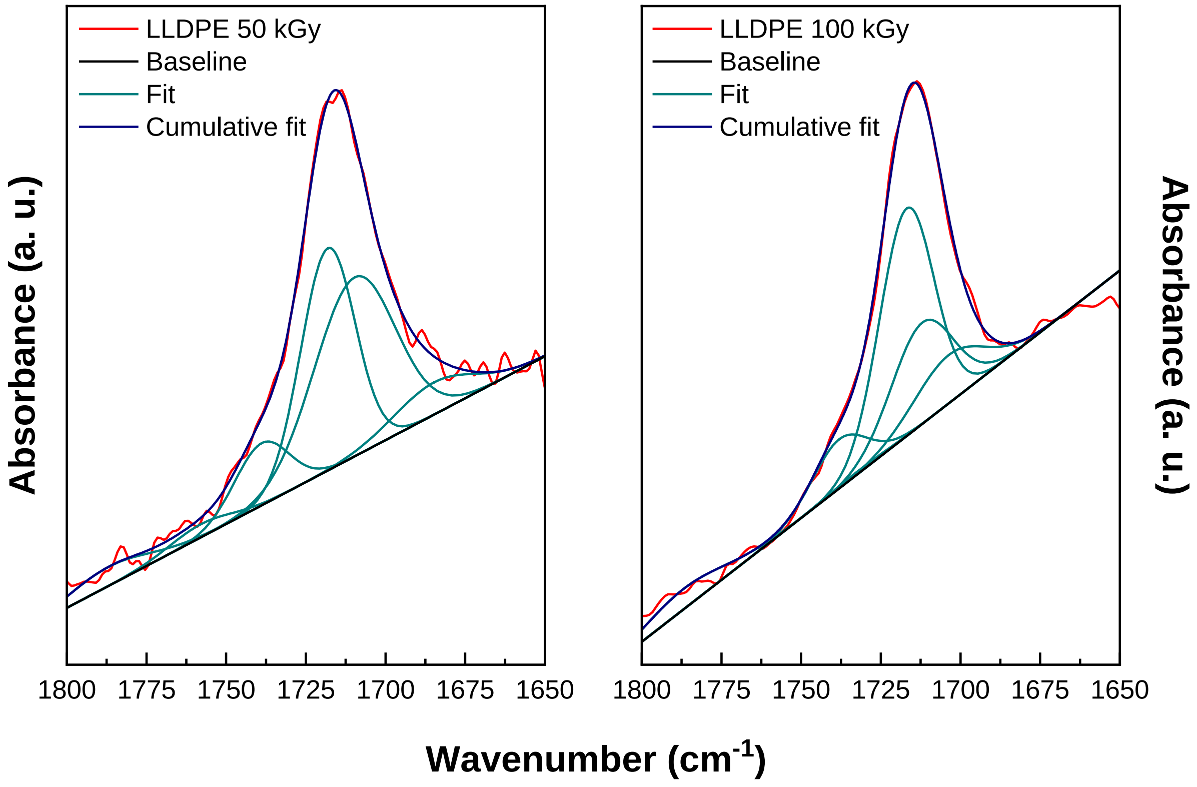

Figure S9. Deconvolution results for LLDPE irradiated at 50 kGy (left) and 100 kGy (right). Peak position parameters are given in the main body of the article. FWHM were constrained between 20 and 30 cm^{-1}. Associated numerical results are given in Table S7.

FTIR band attribution	Wavenumber (cm^{-1})	LLDPE 50 kGy			LLDPE 100 kGy		
		Position	Height	FWHM	Position	Height	FWHM
Lactone	1785.0	1787.0	$1.01\ 10^{-4}$	30	1787.0	$2.64\ 10^{-4}$	29.9
Ketone + alcohol	1705.6	1707.6	$\simeq 0$	30	1703.6	$6.24\ 10^{-4}$	25.9
Free carboxylic acid of peracid	1756.0	1758.0	$6.96\ 10^{-5}$	22.9	1754.0	$\simeq 0$	30
Ester	1740.0	1738.9	$3.30\ 10^{-4}$	20	1738.0	$6.34\ 10^{-4}$	20
Aldehyde	1730.0	1728.0	$\simeq 0$	29.9	1732.0	$3.59\ 10^{-5}$	20
Ketone	1718.0	1718.1	0.00118	20	1716.9	0.00294	20
Carboxylic acid	1710.0	1709.7	$9.51\ 10^{-4}$	29.8	1712.0	0.00131	22.7
Conjugated ketone	1685.0	1684.8	$1.71\ 10^{-4}$	29.8	1687.0	$\simeq 0$	20.1

Table S7. Numerical results of deconvolution for LLDPE irradiated at 50 kGy (left) and 100 kGy (right). Peak position parameters are given in the main body of the article. FWHM were constrained between 20 and 30 cm^{-1}.

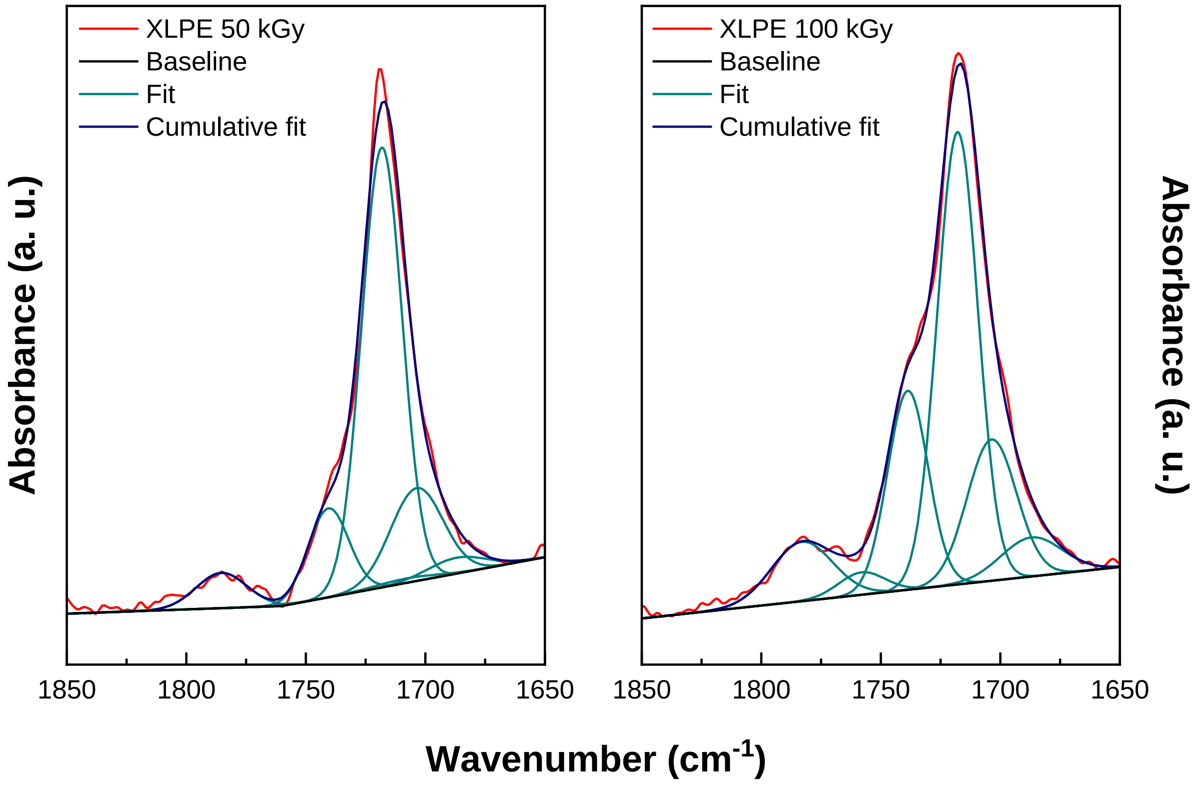

Figure S10. Deconvolution results for XLPE irradiated at 50 kGy (left) and 100 kGy (right). Peak position parameters are given in the main body of the article. FWHM were constrained between 20 and 30 cm^{-1}. Associated numerical results are given in Table S8.

FTIR band attribution	Wavenumber (cm^{-1})	XLPE 50 kGy			XLPE 100 kGy		
		Position	Height	FWHM	Position	Height	FWHM
Lactone	1785.0	*1785.3*	*6.69 10^{-4}*	*25*	1783.0	0.00148	30
Ketone + alcohol	1705.6	*1703.6*	*0.00176*	*25.8*	1703.6	0.00354	24.4
Free carboxylic acid of peracid	1756.0	*1754.0*	*0*	*0*	1758.0	5.62 10^{-4}	22.7
Ester	1740.0	*1740.6*	*0.00169*	*20*	1738.7	0.00498	20
Aldehyde	1730.0	*1729.9*	*0*	*0*	1732.0	$\simeq 0$	30
Ketone	1718.0	*1718.2*	*0.00835*	*20*	1717.9	0.01133	20
Carboxylic acid	1710.0	*1708.0*	*8.40 10^{-5}*	*30*	1708.0	$\simeq 0$	30
Conjugated ketone	1685.0	*1687.0*	*3.00 10^{-4}*	*28.3*	1687.0	9.79 10^{-4}	30

Table S8. Numerical results of deconvolution for XLPE irradiated at 50 kGy (left, in italics as the fit is not fully converged) and 100 kGy (right). Peak position parameters are given in the main body of the article. FWHM were constrained between 20 and 30 cm^{-1}.

2.4. Theoretical wavenumber values with FWHM comprised between 10 and 20 cm^{-1}

Figure S11 presents the deconvolution results obtained for LLDPE irradiated at 50 kGy (left) and 100 kGy (right), peak position parameters being given in the main body of the article and FWHM chosen between 10 and 20 cm^{-1}. Numerical results are given in Table S9.

An equivalent presentation for XLPE irradiated at 50 kGy and 100 kGy is given in Figure S12 and Table S10.

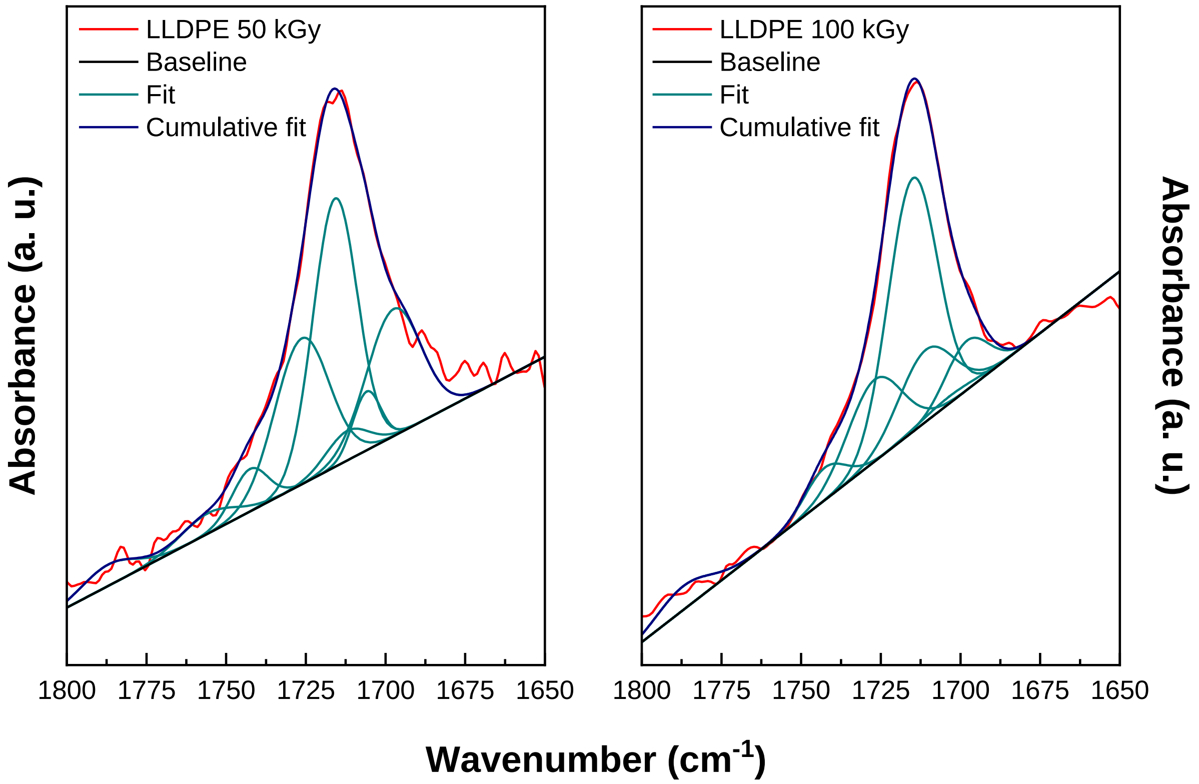

Figure S11. Deconvolution results for LLDPE irradiated at 50 kGy (left) and 100 kGy (right). Peak position parameters were taken from the DFT calculations (scaled by a factor of 1.025). FWHM were constrained between 10 and 20 cm^{-1}. Associated numerical results are given in Table S9.

FTIR band attribution	Wavenumber (cm^{-1})	XLPE 50 kGy			XLPE 100 kGy		
		Position	Height	FWHM	Position	Height	FWHM
H-bonded carboxylic acid	1710	1712.0	$1.57\ 10^{-4}$	15.7	1710.8	$9.28\ 10^{-4}$	20
Lactone	1785	1787.0	$1.14\ 10^{-4}$	20	1787.0	$3.07\ 10^{-4}$	20
Ester	1726.1	1726.3	$7.75\ 10^{-4}$	20	1726.9	0.00106	20
Ketone	1713.8	1715.8	0.00143	16.3	1714.9	0.00327	18.2
Aldehydes	1744.55	1742.6	$2.26\ 10^{-4}$	13.0	1742.6	$4.19\ 10^{-4}$	14.9
Enone	1699.45	1697.5	$6.75\ 10^{-4}$	20	1697.9	$6.34\ 10^{-4}$	15.8
Ketone+alcohol	1705.6	1705.9	$3.12\ 10^{-4}$	10.2	1703.6	$9.15\ 10^{-5}$	20
Free carboxylic acid	1753.77	1755.8	$1.08\ 10^{-4}$	20	1755.8	0	20

Table S9. Numerical results of deconvolution for LLDPE irradiated at 50 kGy (left) and 100 kGy (right). Peak position parameters were taken from the DFT calculations (scaled by a factor of 1.025). FWHM were constrained between 10 and 20 cm^{-1}.

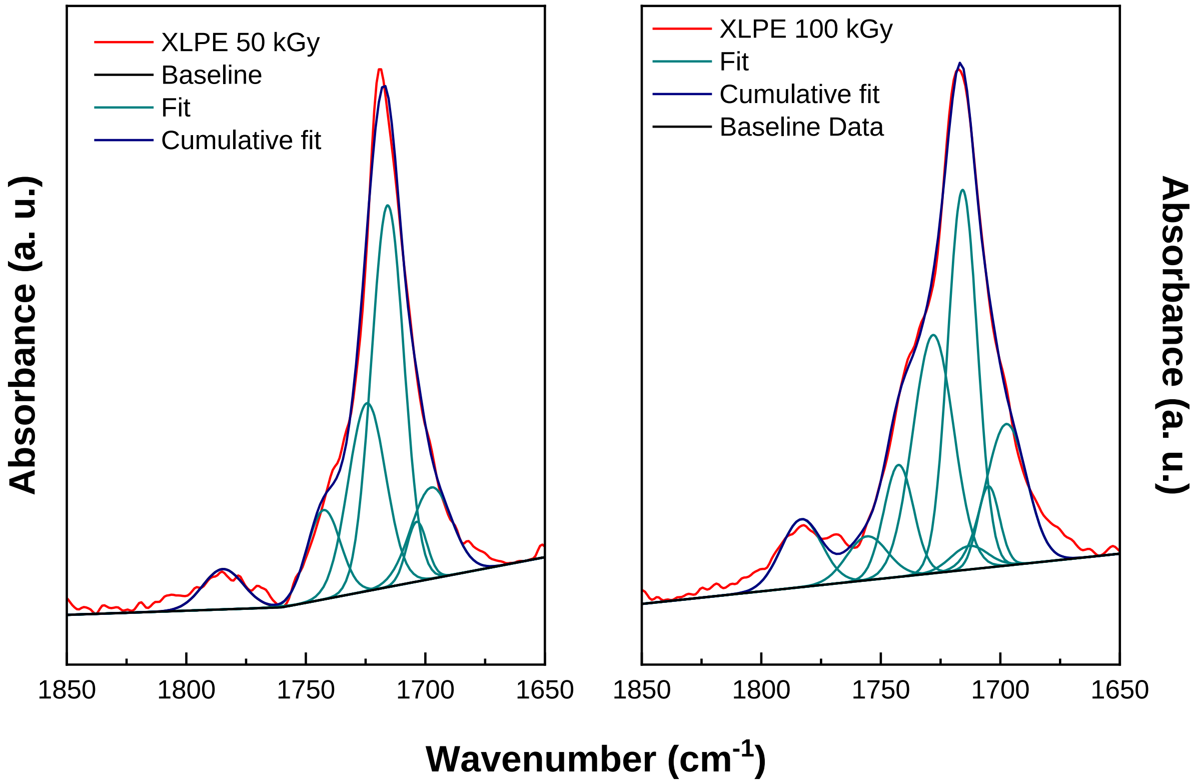

Figure S12. Deconvolution results for XLPE irradiated at 50 kGy (left) and 100 kGy (right). Peak position parameters were taken from the DFT calculations (scaled by factor of 1.025). FWHM were constrained between 10 and 20 cm^{-1}. Associated numerical results are given in Table S10.

FTIR band attribution	Wavenumber (cm^{-1})	XLPE 50 kGy			XLPE 100 kGy		
		Position	Height	FWHM	Position	Height	FWHM
H-bonded carboxylic acid	1710	1712.0	$\simeq 0$	10.3	1713.0	$6.26\ 10^{-4}$	18.0
Lactone	1785	1784.7	$7.66\ 10^{-4}$	20	1783.2	0.0018	20
Ester	1726.1	1724.7	0.00357	18.4	1728.1	0.00633	20
Ketone	1713.8	1715.8	0.00724	15.9	1715.8	0.01010	14.5
Aldehydes	1744.55	1742.6	0.00169	16.4	1742.6	0.00297	14.6
Enone	1699.45	1697.5	0.00173	20	1697.5	0.00376	20
Ketone+alcohol	1705.6	1703.6	0.00113	10	1705.0	0.00216	10.8
Free carboxylic acid	1753.77	1755.8	0	20	1755.8	0.00117	20

Table S10. Numerical results of deconvolution for XLPE irradiated at 50 kGy (left) and 100 kGy (right). Peak position parameters were taken from the DFT calculations (scaled by a factor of 1.025). FWHM were constrained between 10 and 20 cm^{-1}.

2.5. Comparison

Comparison of the adjusted R-Squares is given in Table S11. It evidences that the best numerical adjustment is the one allowing the FWHM comprised between 10 and 20 cm^{-1}.

	FWHM 5 – 15 cm^{-1}	FWHM 10 – 20 cm^{-1}	FWHM 15 – 25 cm^{-1}	FWHM 20 – 30 cm^{-1}	FWHM 10 - 20 cm^{-1} (theoretical values)
**LLDPE 50 kGy**	0.991091	0.993299	0.993890	0.994214	0.987201
**LLDPE 100 kGy**	0.992562	0.993931	0.993200	0.992989	0.992791
**XLPE 50 kGy**	0.991155	0.997991	0.997352	-	0.993315
**XLPE 100 kGy**	0.992933	0.997183	0.998421	0.997685	0.993668

Table S11. R-Square of the different mathematical adjustments of the carbonyl band.

3. Comparison between theoretical and experimental FTIR spectra

3.1. Carbonyl species in small molecules

Figure S13 presents a comparison between experimental and theoretical results for the infrared carbonyl signature in a few small molecules: acetone, acetaldehyde, propionic acid, methyl formate, delta-valerolactone and gamma-butyrolactone, corresponding respectively to ketones, aldehydes, carboxylic acids, esters, and lactones for the last two. The calculations for small molecules were performed in a face-centered cubic unit cell with a cube side of 80 Bohr, 100 Rydberg energy cutoff and the same xc-functional and norm-conserving pseudopotentials as those used for the calculations presented in the main paper.

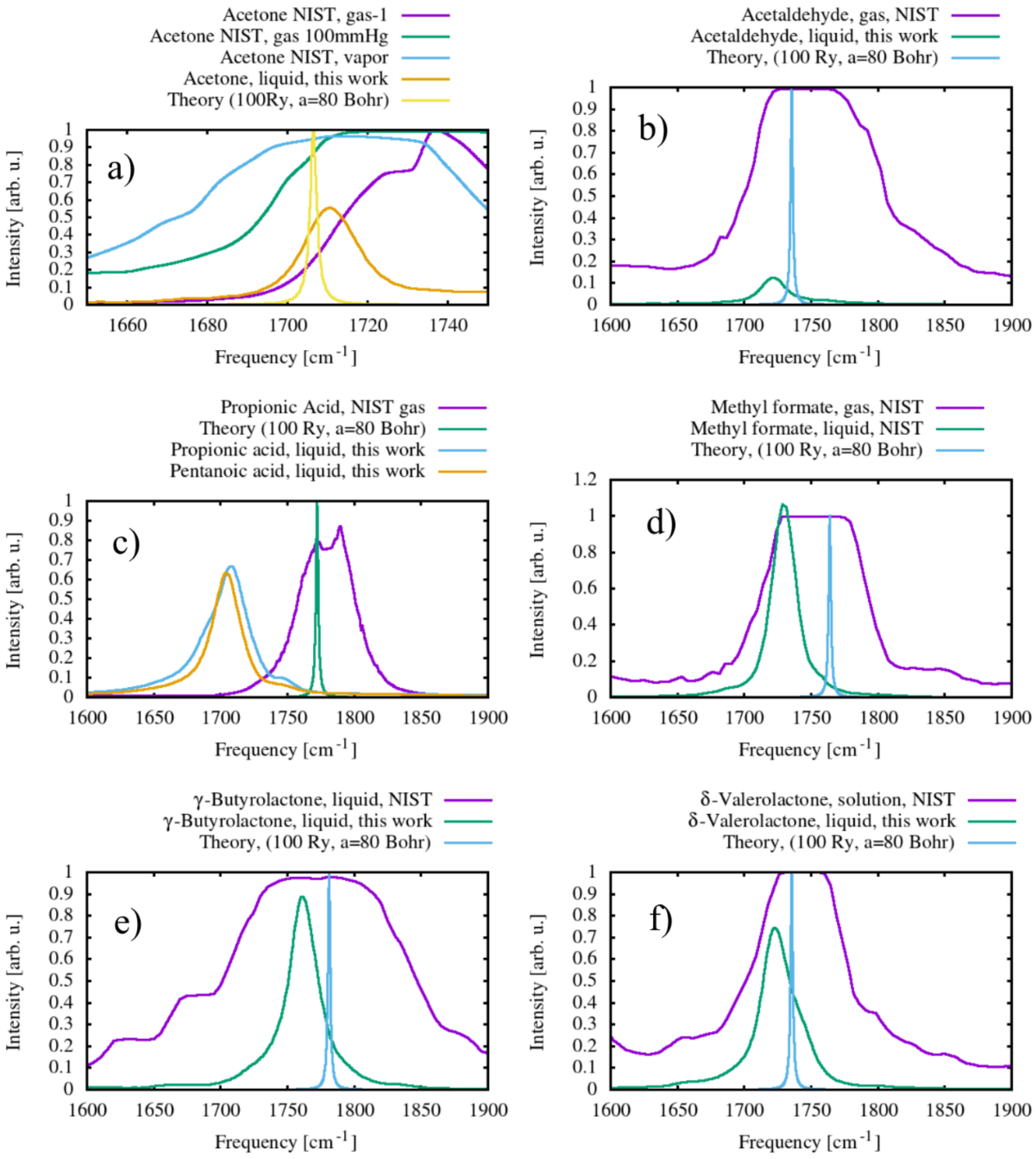

Figure S13. Comparison of experimental and theoretical results for the infrared carbonyl signature in small molecules : a) acetone, b) acetaldehyde, c) propionic acid, d) methyl formate e) γ-butyrolactone, f)δ-valerolactone .

3.2. Virgin Polymers FTIR spectra

Figure S14 presents the comparison of theoretical and experimental spectra of virgin PE. LLDPE and XLPE are the virgin samples on which experiments were performed. The theoretical models are the following: « Lc » is the model used in the main paper, featuring a crystalline lamella and a chain connecting the neighbouring periodic images. The « Lnc » model is like the Lc model but without the connecting chain (empty space between periodic images of the lamellae in the direction perpendicular to them). « Lncc » is similar to Lnc, but with a reduced empty space between lamellae. « (Single) chain » refers to an infinite periodic chain (the dimension of the unit cell in the plane perpendicular to the chain is 20x20 Bohr). « Crystal » indicates the orthorhombic crystal.

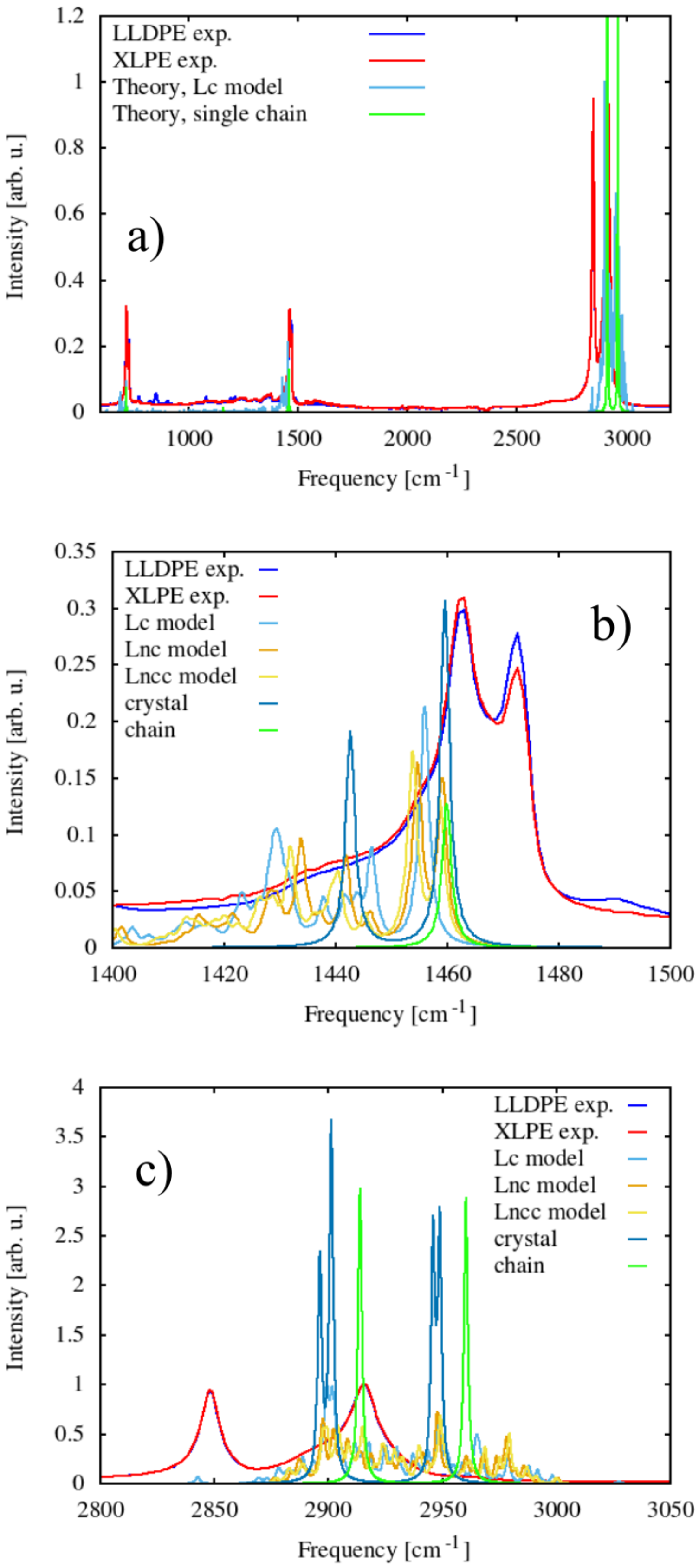

Figure S14. Comparison of theoretical and experimental spectra of virgin PE. a) The full frequency range. b) Zoom on the peaks just below 1500 cm^{-1}. c) Zoom on the high frequency peaks just below 3000 cm^{-1}.

References

Xu, A., Roland, S., Colin, X., 2020. Physico-chemical characterization of the blooming of Irganox 1076® antioxidant onto the surface of a silane-crosslinked polyethylene. Polymer Degradation and Stability 171, 109046.

Hulse, G.E., Kersting, R.J., Warfel, D.R., 1981. Chemistry of dicumyl peroxide-induced crosslinking of linear polyethylene. Journal of Polymer Science: Polymer Chemistry Edition 19, 655-667.

Carlsson, D.J., Brousseau, R., Zhang, C., Wiles, D.M., 1988. Identification of Products from Polyolefin Oxidation by Derivatization Reactions, Chemical Reactions on Polymers. American Chemical Society, pp. 376-389.
